# Chronic Psychological Stress Activates TRP/TAM/CXCL1 Signaling to Promote Breast Cancer Adipocyte Lipolysis via KEAP1 m^6^A Demethylation

**DOI:** 10.34133/research.0980

**Published:** 2025-11-17

**Authors:** Dandan Zhan, Yingqi She, Xinqing Zhang, Juping Zhang, Shengqi Wang, Shicui Hong, Yifeng Zheng, Neng Wang, Zhiyu Wang

**Affiliations:** ^1^ State Key Laboratory of Traditional Chinese Medicine Syndrome, The Second Affiliated Hospital of Guangzhou University of Chinese Medicine, Guangzhou, Guangdong, China.; ^2^ Guangdong-Hong Kong-Macau Joint Lab on Chinese Medicine and Immune Disease Research, Guangzhou University of Chinese Medicine, Guangzhou, Guangdong, China.; ^3^ Guangdong Provincial Key Laboratory of Clinical Research on Traditional Chinese Medicine Syndrome, Guangdong Provincial Academy of Chinese Medical Sciences, Guangdong Provincial Hospital of Chinese Medicine, Guangzhou, Guangdong, China.; ^4^ School of Basic Medical Sciences, Guangzhou University of Chinese Medicine, Guangzhou, Guangdong, China.

## Abstract

Chronic unpredicted mild stress (CUMS) and obesity are well-known risk factors for breast cancer (BC). However, their underlying correlation and potential mechanisms remain unknown. Herein, CUMS was found to promote BC growth and metastasis with increased adipocyte lipolysis, reactive oxygen species (ROS) burst, and mitochondrial fission. Metabolomic analysis identified tryptophan (TRP) as the main responding metabolite, which markedly induced macrophage M2 polarization and CXCL1 expression, and in turn promoted adipocyte lipolysis. Molecular investigation showed that CXCL1 triggered KEAP1 m^6^A demethylation via FTO (fat mass and obesity-associated protein) up-regulation, subsequently reducing NRF2 expression to exacerbate ROS burst and mitochondrial fission. Furthermore, either siCXCL1 or FTO inhibition markedly inhibited CUMS-induced BC progression, accompanied by suppression of adipocyte lipolysis, ROS production, and mitochondrial fission, as well as KEAP1 m^6^A demethylation. Our findings highlight the novel signaling of TRP/TAM/CXCL1 in mediating adipocyte lipolysis underlying CUMS-induced BC progression, and KEAP1 m^6^A demethylation presents a promising therapeutic target.

## Introduction

Breast cancer (BC) ranks first in incidence among female malignancies, accounting for 31% of all cancer types [1–3]. Epidemiological studies have shown that the prevalence of psychiatric disorders among BC patients reached as high as 41.6% [4], with as many as 32.2% and 41.9% of patients suffering from depression and anxiety, respectively [4,5]. A meta-analysis based on 17 randomized controlled trials showed that depression was associated with BC recurrence [1.24 (1.07, 1.43)], all-cause mortality [1.30 (1.23, 1.36)], and tumor-specific mortality [1.29 (1.11, 1.49)], underlining the significance of depression as a cause of cancer recurrence [6]. Currently, more attention has been paid to improve BC patients’ clinical prognosis utilizing both nonpharmacological and pharmacological psychological interventions. Clinical oncologists have demonstrated that they positively reduced BC recurrence and mortality [7]. However, the underlying molecular mechanisms of chronic psychological stress-mediated BC development are awaiting to be clarified. It is important to explore the involved signaling pathway and develop corresponding therapeutic strategies to improve clinical prognosis.

Emerging studies demonstrated that chronic psychological stress could increase the secretion of adrenaline, noradrenaline, glucocorticoids, prolactin, and oxytocin, accompanied by decreased dopamine secretion [8]. It is believed that the level of 5-hydroxytryptamine (5-HT), a biologically active neurotransmitter derived from tryptophan (TRP) metabolism, was closely correlated with depression [9]. 5-HT is mainly converted from TRP by hydroxylase in the central nervous system, as well as in gastrointestinal enterochromaffin cells [10]. 5-HT receptors and their downstream signaling have been found closely correlated with cancer growth and metastasis [11]. TRP is mainly metabolized through 3 pathways, leading to the synthesis of kynurenine, 5-HT, and indole derivatives [12]. TRP metabolism imbalance is recorded with neurological and psychiatric dysfunctions, chronic immune activation, and immune suppression microenvironment in malignancies [13]. However, the important contribution of TRP in mediating chronic psychological stress-induced cancer development still remains unknown. Hence, it is imperative to explore the effects of TRP on BC microenvironment, particularly for the adipocytes, which constitute more than 80% in breast tissues.

In the BC microenvironment, adipocytes interact with cancer cells to form a vicious cycle that promotes tumor proliferation, metastasis, and drug resistance [14]. Adipocyte lipolysis is the catabolism of triacylglycerols stored in cellular lipid droplets [15], providing fatty acids to other organs during energy-demand periods [16]. Notably, a close relationship exists between psychological stress and adipocyte metabolism. Under stress conditions, an increase of circulating noradrenaline could induce lipolysis and enhance free fatty acids (FFAs) releasing into the circulation [17]. These FFAs are subsequently taken up by cancer cells and contribute to cancer growth through the mechanism of mitochondrial fatty acid oxidation (FAO) [18]. Redox signaling is one of the essential mechanisms involved in lipolysis. Reactive Oxygen Species (ROS) is an effector of redox signaling; the uncontrolled ROS production could facilitate lipolysis through the activation of β-adrenergic receptors, adenylyl cyclase, and protein kinase [19]. Furthermore, oxidative stress leads to excessive mitochondrial fission [20]. In brown adipose tissue, mitochondrial fission is considered as a compensatory mechanism to increase FAO [21]. However, the underlying molecular mechanisms of oxidative stress-induced mitochondrial fission in lipolysis still need to be explored.

The crown-like structure (CLS) of the breast is composed of macrophages clustered around hypertrophied, dead, and dying adipocytes, and is considered as a manifestation of adipose tissue inflammation [22]. Generally, CLS is identified by CD68 immunohistochemical staining [23]. Moreover, the CD68^+^CD163^+^-CLS has been reported to exist in adipose tissue around BC lesions, and a high level of CD163^+^ macrophages (tumor-associated macrophages [TAMs]) was linked to shorter DFS (disease-free survival) in node-negative BC patients (*P* = 0.033) [24]. A recent meta-analysis also suggested that high-density TAMs are associated with poor cancer prognosis, and the risk ratios (hazard ratios, 95% confidence interval) for high-density TAMS and low-density TAMS were 1.5 (1.20 to 1.88) and 2.2 (1.72 to 2.89), respectively [25]. Chemokines have been considered as crucial signal transducers secreted by TAMs. The highly expressed chemokines of TAMs include CCL2, CCL3, CCL5, CCL18, CXCL1, and CXCL12 [26]. Several chemokines have been reported to be associated with lipolysis, such as CCL2, CCL5, and CXCL1 [27,28]. CXCL1 has been identified as the highest chemokine secreted by TAMs [26]. Elevated CXCL1 was associated with enlarged tumors, high-grade malignancies, and shortened survival time in BC [29]. Interestingly, Zhang et al. [30] demonstrated that CXCL1 could recruit adipocyte stem cells to promote cancer progression. In addition, CXCL1 was also shown to reduce the fat pads [27]. However, whether TAMs/CXCL1 signaling is involved in chronic stress-induced adipocyte lipolysis remains to be clearly elucidated.

Herein, we demonstrated that chronic psychological stress activated adipocyte lipolysis and mitochondrial fission in BC, and metabolomic analysis validated TRP as the main differential metabolite. TRP could activate adipocyte lipolysis via enhancing TAMs/CXCL1 signaling to induce *KEAP1* m^6^A demethylation, and subsequently inhibit NRF2 expression to trigger mitochondrial fission and lipolysis via ROS burst. CXCL1 silencing or demethylation inhibition suppressed chronic psychological stress-triggered lipolysis and BC development via blocking *KEAP1* m^6^A demethylation. Our results highlight the significant role of psychological stress in mediating adipocyte lipolysis during BC development, and provide TRP/TAM/CXCL1 as the novel signaling for activating adipocyte lipolysis via enhancing *KEAP1* m^6^A demethylation.

## Results

### CUMS promotes BC growth and metastasis

**Fig. 1.**
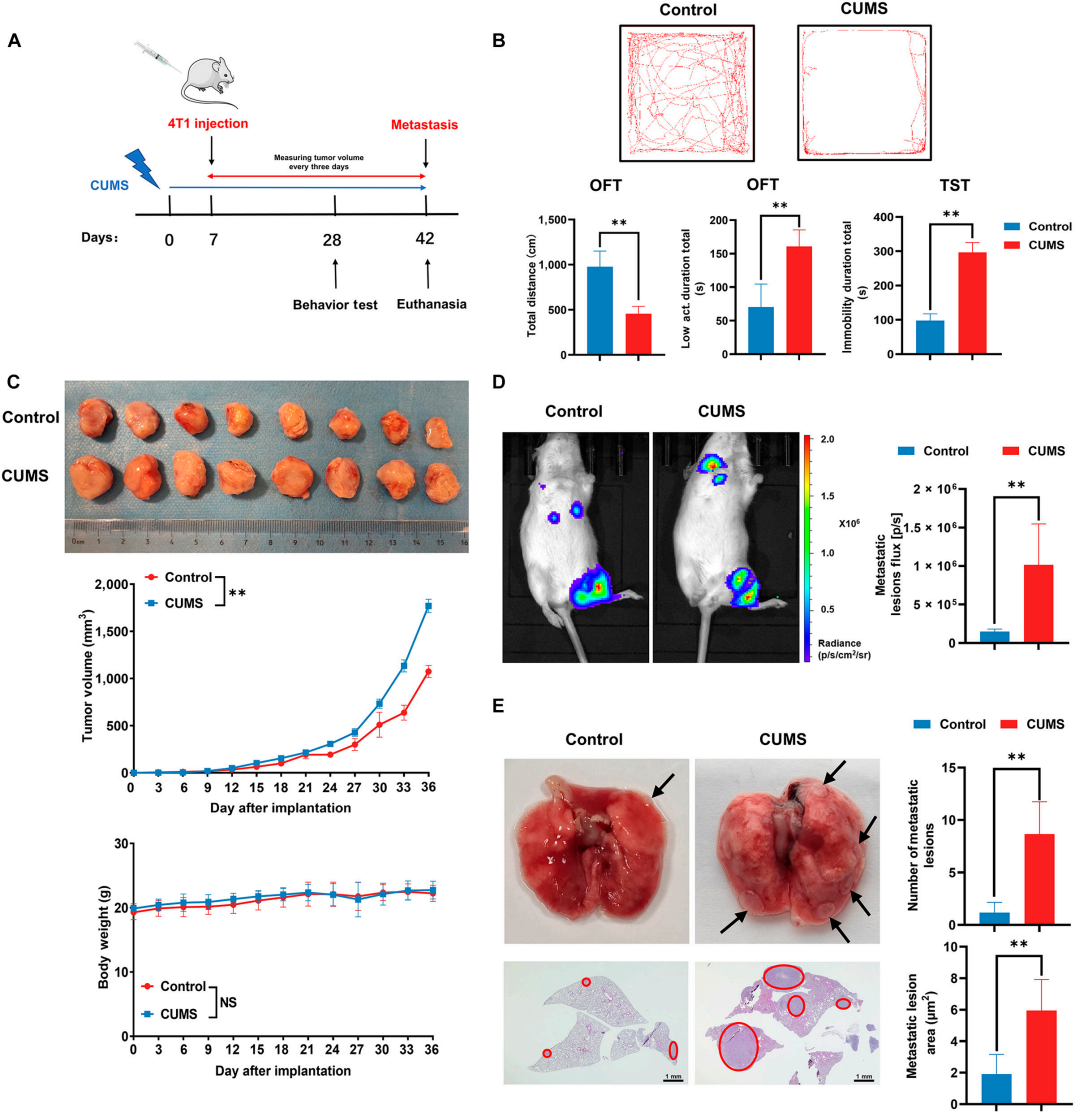
CUMS promotes BC growth and metastasis. (A) Flowchart of the mouse experiments. (B) Behavioral analysis of OFT and TST (*n* = 8). (C) Representative pictures, tumor growth curves, and body weight changes in each group (*n* = 8). (D) The lung metastasis of BC xenograft was detected by the in vivo imaging assay (*n* = 8). (E) Representative images of lung metastasis lesions in gross (*n* = 6) and HE staining (*n* = 8). The arrows and red circles indicate the metastatic tumor foci in murine lungs. Scale bar = 1 mm. Data are presented as mean ± SD, *n* = 8. Statistical analysis: repeated-measures ANOVA for (C) and Student’s *t* test for 2-group comparisons. Data in the top panel of (E) were analyzed by the Mann–Whitney *U* test. **P ≤ 0.01.

Chronic unpredicted mild stress (CUMS) is the most commonly available, reliable, and effective rodent model of depression. Therefore, we exposed mice to CUMS, and subsequently established orthotopic BC mice model by injecting 4T1 cells into mammary fat pads to study the effects of CUMS on BC growth and metastasis (Fig. 1A). The behavioral tests were conducted on the 28th day after the initiation of CUMS. According to the results, CUMS notably decreased the total traveled distance, enhanced the low activity duration in the open field test (OFT), and prolonged the immobility duration in the tail suspension test (TST) (Fig. 1B). These findings suggested the successful establishment of the CUMS model. Meanwhile, CUMS substantially increased BC growth while having little influence on mice body weight (Fig. 1C). Notably, as revealed by the in vivo luciferase imaging assay, CUMS facilitated BC lung metastasis (Fig. 1D). Additionally, according to ex vivo lung observation findings, CUMS increased metastatic nodule numbers in the lung, accompanied by increased metastatic areas detected by hematoxylin and eosin (HE) staining (Fig. 1E). In summary, CUMS may facilitate BC growth and metastasis.

### CUMS activates lipolysis of pericancerous adipose tissue of BC

**Fig. 2.**
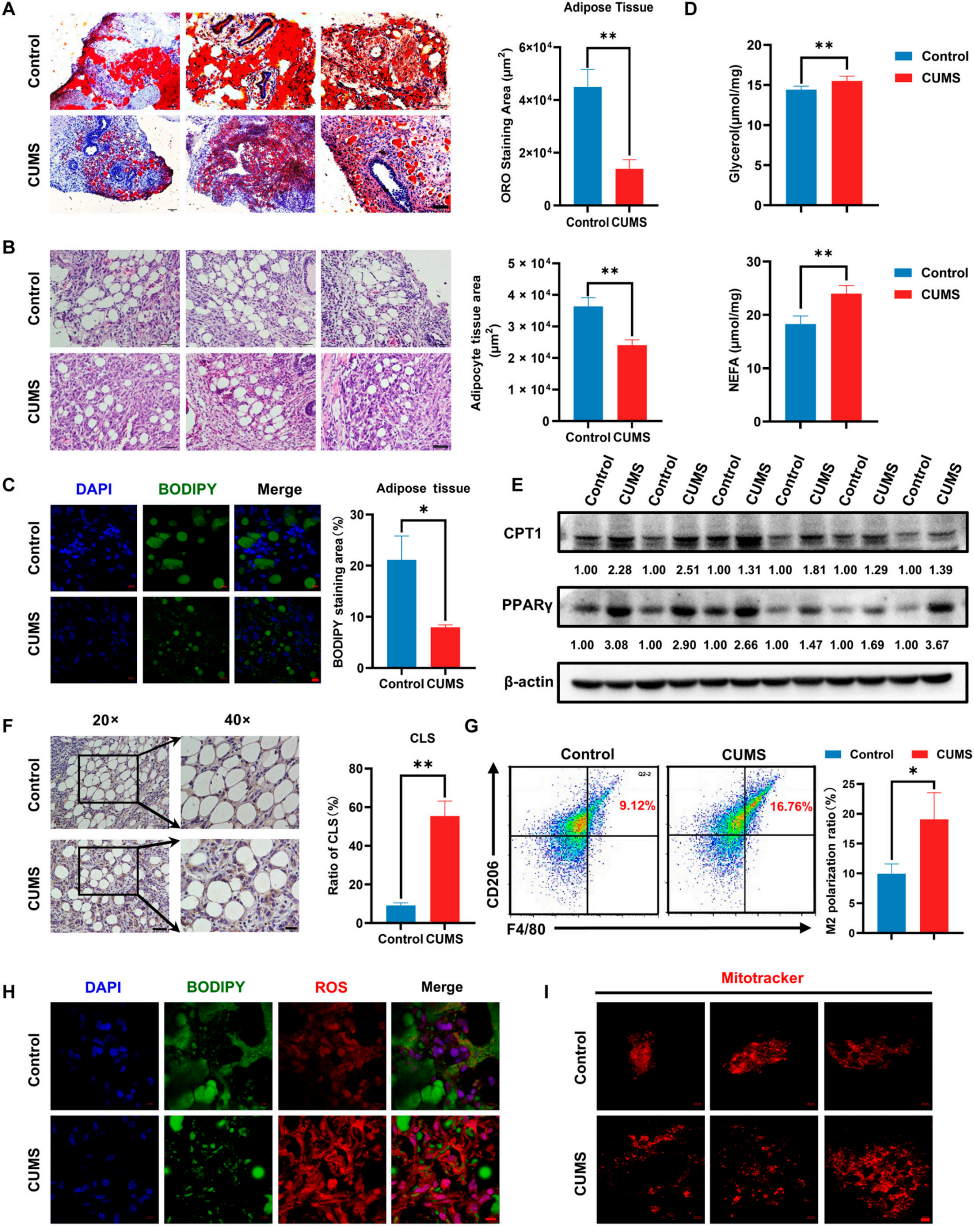
Pathological changes of adipose tissue nearby BC by CUMS. (A and B) Representative Oil Red O staining (A) and HE caused staining (B) in adipose tissue nearby BC in different groups (*n* = 6). Scale bar = 50 μm. (C) Representative BODIPY staining in adipose tissue nearby breast tumor (*n* = 3). Scale bar = 10 μm. (D) Glycerol and NEFA levels in adipose tissue nearby BC (*n* = 6). (E) The fatty acid oxidation-related protein expression levels in tumor tissue were detected by Western blotting (*n* = 6). (F) Photomicrographs of crown-like structures in the adipose tissue nearby breast tumor of different groups, shown by immunohistochemistry staining with anti-CD68, a macrophage marker (*n* = 6). 20×: Scale bar = 50 μm. 40×: Scale bar = 20 μm. (G) The infiltration level of M2-like macrophages in adipose tissue nearby BC was detected by flow cytometry (*n* = 3). (H) Representative ROS staining in adipose tissue nearby BC in different groups (*n* = 3). Scale bar = 10 μm. (I) Representative MitoTracker staining in primary adipocytes nearby BC in different groups (*n* = 3). Scale bar = 10 μm. Data are presented as mean ± SD, Student’s *t* test for 2-group comparisons. *P ≤ 0.05, **P ≤ 0.01.

The pericancerous adipose tissue has been validated as a crucial pro-cancer factor in multiple malignancies [31]. In the CUMS-treated BC model, Oil Red O staining also revealed notably smaller lipid droplets in adipocytes adjacent to cancer cells compared to those in the control group (Fig. 2A). HE staining also demonstrated that pericancerous adipocytes were smaller in the CUMS group (Fig. 2B). Moreover, boron-dipyrromethene (BODIPY) staining further validated that CUMS resulted in smaller lipid droplets in the para BC adipocytes (Fig. 2C). To investigate the metabolism changes of adipocytes responding to CUMS, we detected the concentrations of nonesterified fatty acid (NEFA) and glycerol in the pericancerous adipose tissues. The results revealed that the concentrations of NEFA and glycerol in the adipose tissue were markedly elevated following CUMS administration (Fig. 2D). Meanwhile, the expression of FAO-related proteins CPT1 and peroxisome proliferator-activated receptor γ (PPARγ) was remarkably increased in the primary adipocytes following CUMS treatment (Fig. 2E). Therefore, CUMS may promote BC growth via activating lipolysis. It has been reported that CLS is closely correlated with adipocyte lipolysis [32]. Therefore, we detected the changes of CLS by immunohistochemistry staining. The results showed that CUMS induced a remarkable increase in the number of CLS around cancer tissue (Fig. 2F). Subsequently, flow cytometry analysis indicated that the level of M2-like macrophage infiltration was higher in the CLS of the CUMS group (Fig. 2G). Since oxidative stress and mitochondrial fission have been known to play a crucial role in the process of adipocyte lipolysis, we further examined ROS level and mitochondrial fission status in pericancerous adipose tissue. The results showed that CUMS elevated ROS levels and promoted the increase of punctate mitochondria when compared to the control group (Fig. 2H and I). These findings validate that CUMS substantially triggers adipocyte lipolysis, which is closely correlated with ROS production and mitochondrial fission.

### CUMS inhibits TRP metabolism

**Fig. 3.**
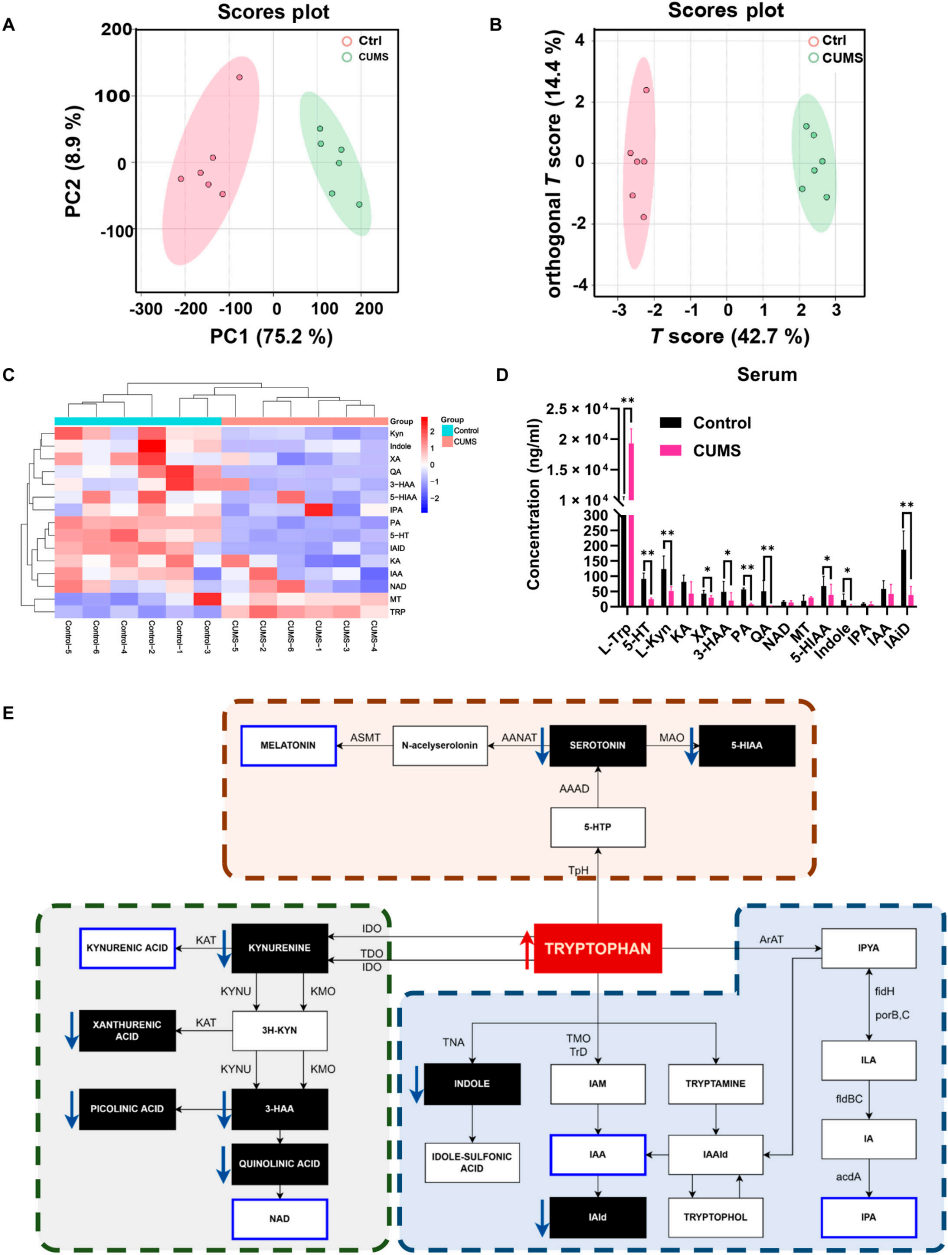
Effect of CUMS on TRP metabolism in BC-bearing mouse serum. (A and B) PCA and OPLS-DA plot of global serum samples. (C) Heatmap depicting the average metabolite intensities in the metabolomics data. (D) The content changes of 15 tryptophan metabolisms in serum. (E) Schematic illustration depicting TRP metabolism alterations induced by CUMS in serum. Data are presented as mean ± SD, *n* = 6. Student’s *t* test for 2-group comparisons. Data of the 3-HAA, PA, QA, MT, 5-HIAA, Indole, and IAid in (D) were analyzed by the Mann–Whitney *U* test. *P ≤ 0.05, **P ≤ 0.01.

Current evidence suggests that depression is closely associated with dysregulated TRP metabolism [33]. In the host organism, there are 3 major TRP metabolic pathways that are responsible for the generation of serotonin, kynurenine, and indole derivatives [12]. To determine the effect of CUMS on TRP metabolism, the UPLC-MS/MS detection was employed for the TRP metabolism pathway including 15 metabolites. The unsupervised principal component analysis (PCA) scatter plot was used to show the different metabolic trends between 2 groups. As indicated by the results, the TRP metabolites of the CUMS group were clearly separated from the control group (Fig. 3A). Meanwhile, the supervised orthogonal projections to latent structures discriminant analysis (OPLS-DA) also demonstrated that CUMS induced a significant metabolite profile change (Fig. 3B). To better assess the effects of CUMS on TRP metabolites, a total of 15 metabolites were quantified, and agglomerative hierarchic cluster analysis was conducted. It revealed a significant metabolism alteration in CUMS-treated mice (Fig. 3C). Moreover, the metabolites in the TRP metabolic pathway were compared between groups, and the results showed that CUMS markedly increased TRP level in mouse serum. However, the levels of other metabolites including 5-HT, L-kynurenine, xanthurenic acid, 2-amino-3-hydroxybenzoic acid, 2-picolinic acid, quinolinic acid, 5-hydroxyindoleacetic acid, indole, and indole-3-carboxaldehyde were decreased following CUMS administration (Fig. 3D). Finally, the TRP metabolic pathway was summarized in Fig. 3E, and TRP was identified as the key elevated metabolite responding to CUMS treatment.

### TRP promotes macrophage polarization and CXCL1-mediated adipocyte lipolysis

To further explore whether TRP could induce macrophage polarization and adipocyte lipolysis, we treated macrophages with TRP in vitro. Flow cytometric analysis revealed that TRP induces M2 macrophage polarization in a dose-dependent manner (Fig. S1A), concomitantly inhibiting M1 macrophage polarization (Fig. S1B), suggesting a transition to an anti-inflammatory phenotype that facilitates tumor progression. To identify the molecular mechanism by which macrophages sense TRP, we employed CH-223191, a specific aryl hydrocarbon receptor (AhR) antagonist. Pretreatment with CH-223191 substantially reversed TRP-induced M2 polarization while restoring M1 phenotype markers (Fig. S1C and D). Furthermore, while TRP dose- and time-dependently increased CXCL1 secretion and expression, CH-223191 pretreatment markedly attenuated these effects (Fig. S1E to I). These findings identify AhR as the principal receptor that mediates the immunomodulatory effects of TRP on macrophages and the subsequent production of CXCL1.

**Fig. 4.**
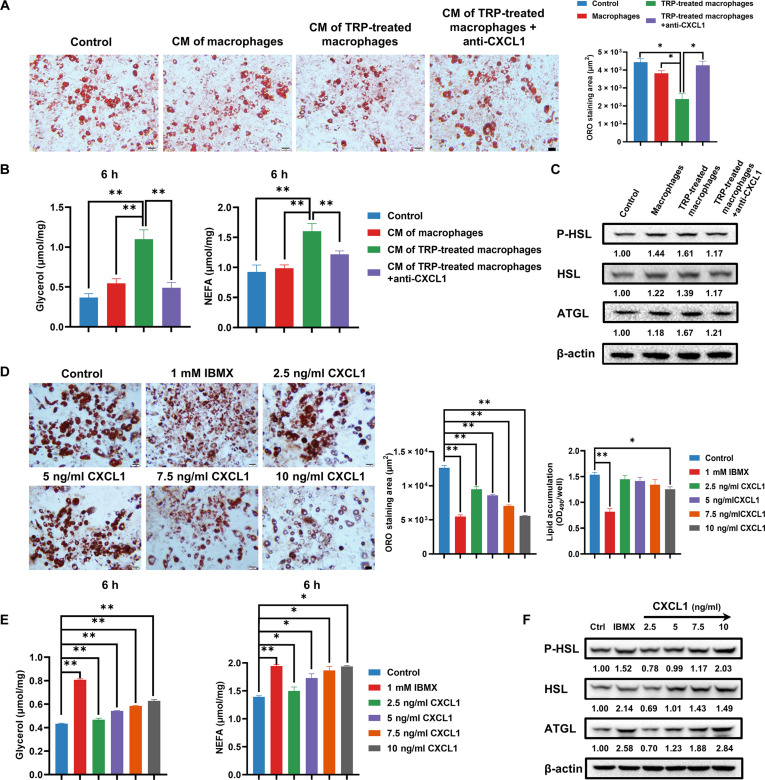
TRP promotes macrophage polarization and induces adipocyte lipolysis via CXCL1. (A) Representative Oil Red O staining images of adipocytes administered with indicated treatments after 48 h. (B) The glycerol and NEFA levels in culture medium after 6 h treatment as indicated. (C) The lipolysis-related protein expression levels were detected by Western blotting after 24 h treatment as indicated. (D) Representative Oil Red O staining images of adipocytes treated with increasing CXCL1 dosage after 48 h. (E) The glycerol and NEFA levels in culture medium after CXCL1 treatment for 6 h. (F) The lipolysis-related protein expression levels were detected by Western blotting after CXCL1 treatment for 24 h. Scale bar = 50 μm. Data are presented as mean ± SD, *n* = 3. Statistical analysis: one-way ANOVA with Bonferroni post-hoc test for (A) and (B), Dunnett *t* post-hoc test for (D) and (E). *P ≤ 0.05, **P ≤ 0.01.

To evaluate the influence of TRP on adipocyte lipolysis, the conditional medium of TRP-treated macrophages (TRP-CM) was collected and co-cultured with adipocytes. The oil-red assay showed that TRP-CM markedly reduced the size of lipid droplets, which was blocked following the addition of the CXCL1-neutralizing antibody (NA) (Fig. 4A). Meanwhile, the levels of glycerol and NEFA were also remarkably increased after TRP-CM treatment for 6 h**,** and CXCL1 NA administration also interrupted this process (Fig. 4B). Furthermore, the expression levels of lipolytic proteins such as P-HSL, HSL, and ATGL were elevated after TRP-CM treatment in adipocytes, but were recovered by CXCL1 NA (Fig. 4C). Therefore, we assumed that CXCL1 secreted by macrophages might promote adipocyte lipolysis. To test this hypothesis, adipocytes were directly treated with CXCL1. The results revealed that CXCL1 led to a decrease in lipid droplets, an increase in glycerol and FFA concentrations, and an up-regulation of lipolytic protein levels (Fig. 4D to F). Notably, ATGLi, a specific lipolysis inhibitor, resulted in a reduction of CXCL1-induced effects (Fig. S2).

Moreover, we also demonstrated the effects of CXCL1-mediated lipolysis on BC cell proliferation, invasion, and fatty acid metabolism. IBMX was used as a positive drug to promote lipolysis. The results showed that the colony formation, migration, and invasion of 4T1 cells were all markedly increased following treatment with the CM of CXCL1-treated adipocytes or IBMX (Fig. S3A to C). In addition, the oxygen consumption rate (OCR) was measured using an extracellular flux analyzer. As shown in Fig. S3D, the OCR parameters of 4T1 cells were elevated by the CM of CXCL1-treated adipocytes compared to the control, suggesting that CXCL1-mediated adipocyte lipolysis could enhance the activity of mitochondrial oxidative phosphorylation chain, which facilitated BC growth and metastasis. However, ATGLi reversed these effects of CXCL1-mediated lipolysis (Fig. S4).

### CXCL1 elevates oxidative stress during lipolysis in adipocytes by inhibiting Nrf2

**Fig. 5.**
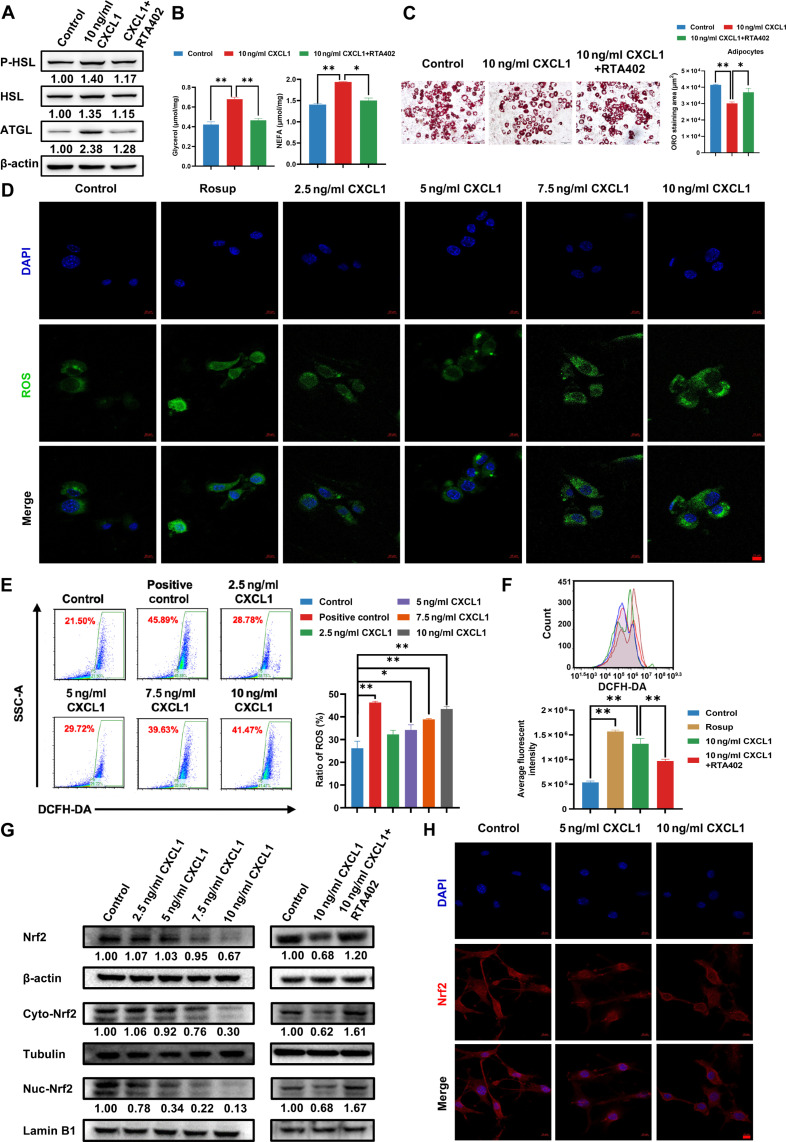
Effect of CXCL1 on the level of oxidative stress in adipocytes. (A) The lipolysis-related protein expression levels were detected by Western blotting following CXCL1 treatment with or without RTA402 after 24 h. (B) The glycerol and NEFA levels in culture medium following CXCL1 treatment with or without RTA402 after 6 h. (C) Representative Oil Red O staining images of adipocytes administered with indicated treatments after 48 h. Scale bar = 50 μm. (D to F) The level of ROS in adipocytes were detected by immunofluorescence assay (D) and flow cytometry assay (E and F) after 24 h CXCL1 with or without RTA402 treatment. (G) The oxidative stress-related protein expression levels in adipocytes were detected by Western blotting after 24 h of CXCL1 with or without RTA402 treatment. (H) Representative immunofluorescence images of NRF2 expression in adipocytes after 24 h of CXCL1 treatment. Scale bar = 10 μm. Data are presented as mean ± SD, *n* = 3. Statistical analysis: one-way ANOVA with LSD post-hoc test for (B) and (C) and Dunnett *t* post-hoc test for (E) and (F). *P ≤ 0.05, **P ≤ 0.01.

We next explored the mechanism by which CXCL1 promotes lipolysis. First, we verified whether CXCL1-mediated lipolysis is induced via oxidative stress. RTA402, an NRF2 agonist, was applied to treat adipocytes along with CXCL1. The expression levels of lipolytic proteins, the levels of glycerol and NEFA, and the oil-red assay showed that RTA402 substantially blocked the effect of CXCL1-mediated lipolysis (Fig. 5A to C). Next, the effect of CXCL1 on ROS levels in adipocytes was determined utilizing immunofluorescence and flow cytometry. It was shown that CXCL1 notably elevated the level of ROS in adipocytes in a dose-dependent manner (Fig. 5D and E). However, this ROS-triggering effect of CXCL1 was relieved by RTA402 (Fig. 5F). Therefore, the modulatory effect of CXCL1 on NRF2, a key regulator of oxidative stress, was studied. Immunoblotting analysis showed that CXCL1 inhibited the expression level of NRF2 in adipocytes. More importantly, the cytoplasmic and nuclear fractionation assay showed that CXCL1 resulted in the reduction of NRF2 expression in both cytoplasm and nucleus of cells. In contrast, RTA402 recovered NRF2 level when combined with CXCL1 administration (Fig. 5G). Meanwhile, immunofluorescence also revealed that CXCL1 inhibited NRF2 expression in adipocytes, either in the cytoplasm or in the nucleus (Fig. 5H). In conclusion, these findings suggest that CXCL1 may promote adipocyte lipolysis via triggering ROS production by NRF2 suppression.

### CXCL1 promotes mitochondrial fission in adipocytes

**Fig. 6.**
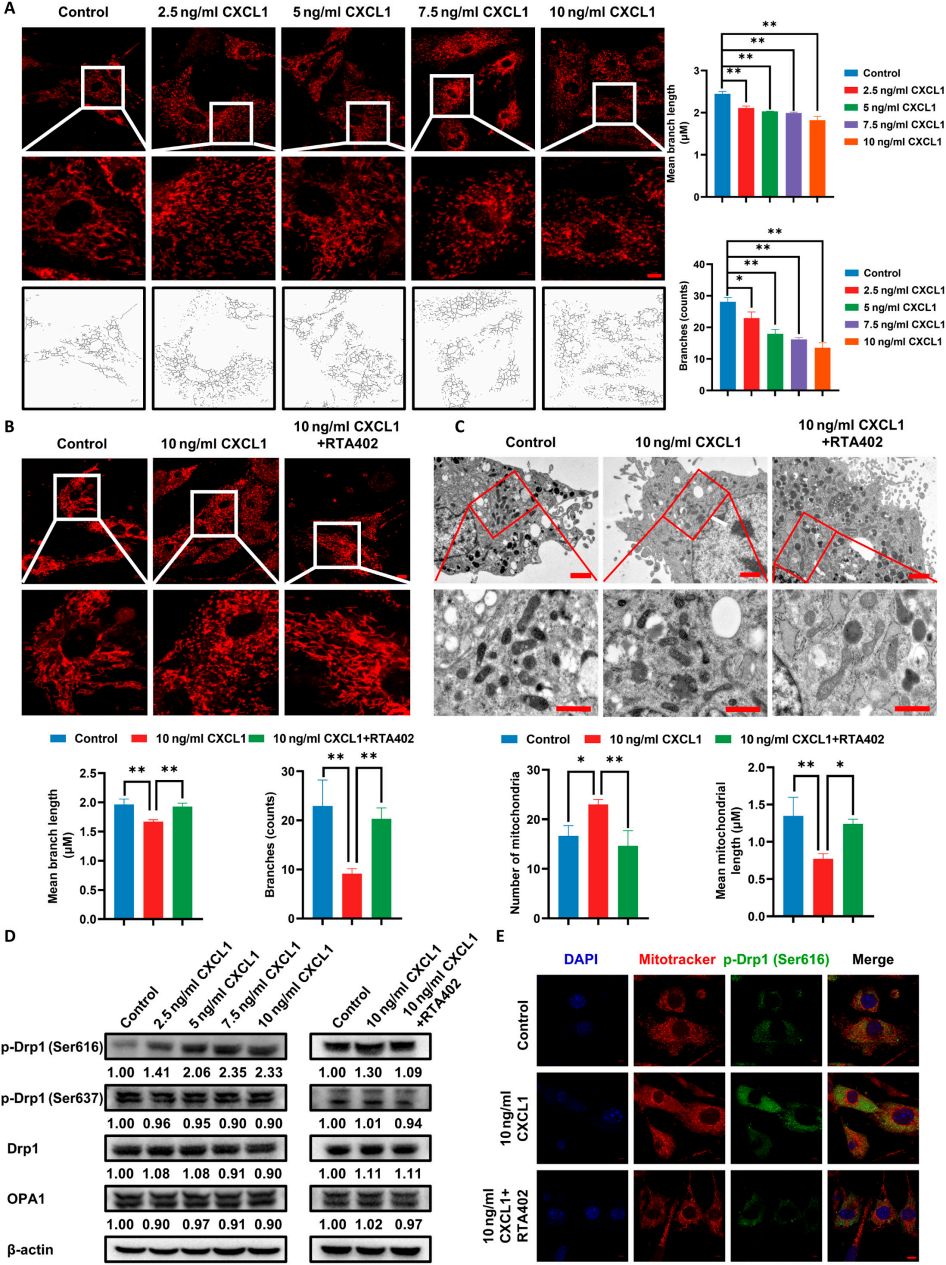
Effect of CXCL1 on mitochondrial fission in adipocytes. (A and B) Representative immunofluorescence images of mitochondrial fission in adipocytes after 24 h of CXCL1 with or without RTA402 treatment. The scale bars indicate 10 and 5 μm, respectively. (C) Mitochondrial fission in adipocytes was photographed by electron microscopy after 24 h of CXCL1 with or without RTA402 treatment. The scale bars indicate 2 and 1 μm, respectively. (D) The mitochondrial fission-associated protein levels in adipocytes were detected by Western blotting after 24 h of CXCL1 with or without RTA402 treatment. (E) Representative immunofluorescence images of p-Drp (Ser616) expression in adipocytes following CXCL1 treatment with or without RTA402 after 24 h. Scale bar = 10 μm. Data are presented as mean ± SD, *n* = 3. Statistical analysis: one-way ANOVA with Dunnett *t* post-hoc test for (A) and LSD post-hoc test for (B) and (C). *P ≤ 0.05, **P ≤ 0.01.

ROS is considered as a crucial factor inducing mitochondrial fission, which is highly implicated in adipocyte lipolysis. Therefore, we attempted to investigate the changes of mitochondrial dynamics during CXCL1-promoted oxidative stress and lipolysis in adipocytes. As shown in Fig. 6A, the cellular immunofluorescence assay found that CXCL1 promotes mitochondrial fission in a dose-dependent manner. However, RTA402 partially reversed this effect detected by MitoTracker staining and transmission electron microscopy (Fig. 6B and C). Moreover, Western blotting proved a dose-dependent up-regulation of mitochondrial fission protein p-Drp1 (Ser616) induced by CXCL1 in adipocytes, while the expression levels of mitochondrial fission protein p-Drp1 (Ser637), Drp1, and mitochondrial fusion OPA1 were not substantially affected. In contrast, p-Drp1 (Ser616) level was down-regulated by RTA402 (Fig. 6D). Furthermore, cellular immunofluorescence results also showed that CXCL1 treatment resulted in short rod-shaped mitochondrion, accompanied by up-regulation of p-Drp1 (Ser616) (Fig. 6E). The above results indicate that CXCL1 promotes mitochondrial fission in adipocytes.

### CXCL1 promotes adipocyte m^6^A demethylation

**Fig. 7.**
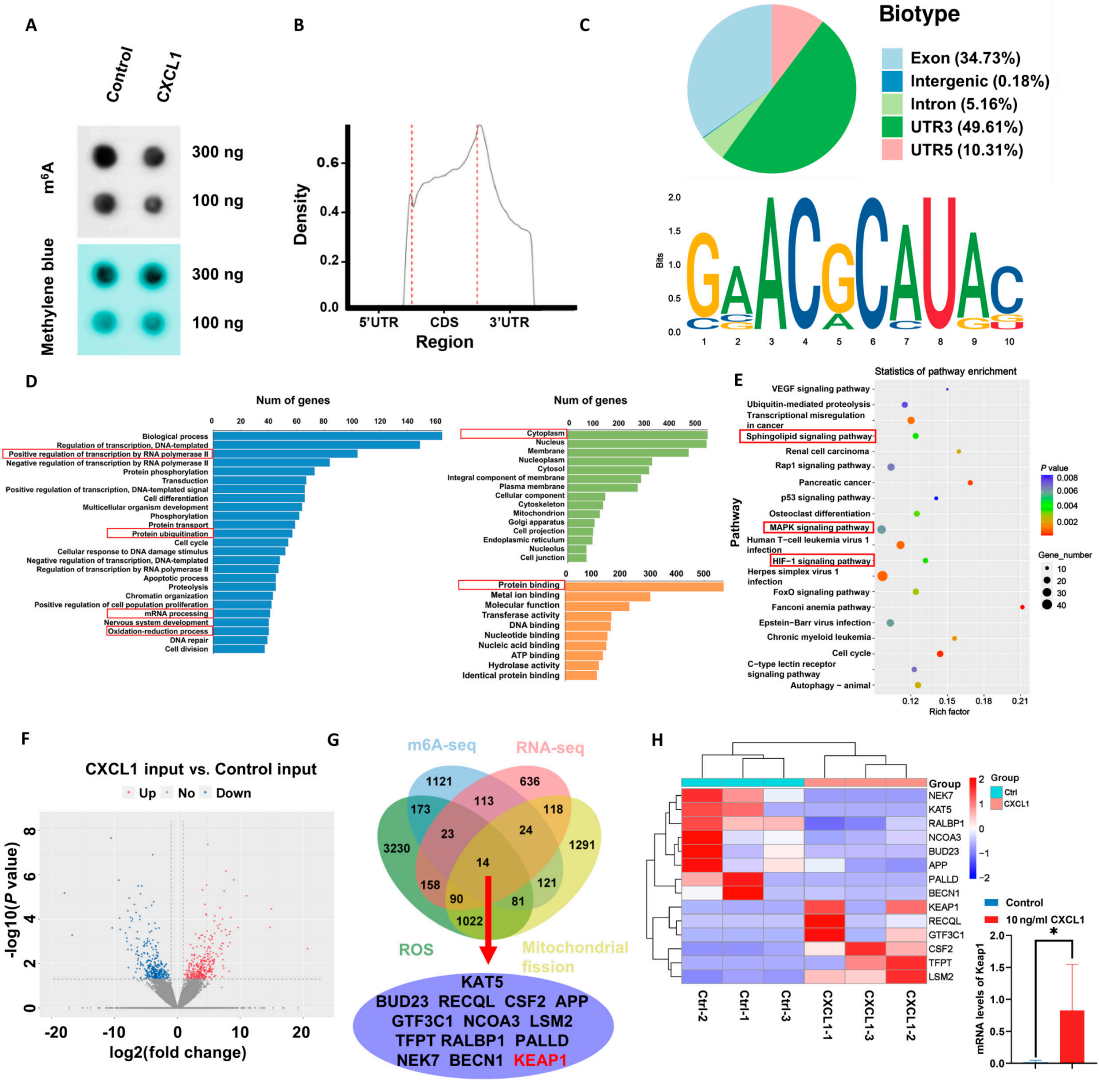
Effect of CXCL1 on m^6^A methylation in adipocytes. (A) The m^6^A dot blot assay of global m^6^A abundance in adipocytes of different groups. (B) Distribution of m^6^A peaks across mRNA transcripts. (C) Graphs of m^6^A peak distribution illustrating the proportion of common m^6^A peaks in the indicated regions (top panel) and HOMER motif analysis revealed the top consensus m^6^A motif in adipocytes (bottom panel). (D) Gene ontology terms identified substantially different gene clusters after CXCL1 intervention in adipocytes. (E) KEGG analysis showed the involved signaling pathways following CXCL1 treatment in adipocytes. (F) Volcano plot analysis of significant mRNA changes occurred in CXCL1-treated adipocytes compared to the control (blue: hypomethylated transcripts; red: hypermethylated transcripts). (G) Venn diagram illustrated overlapping genes among m^6^A-related differential genes, mRNA-related differential genes, ROS-related genes, and mitochondrial fission-related genes. (H) Heatmap analysis of 14 overlapping gene transcripts. Data are presented as mean ± SD, *n* = 3. Student’s *t* test for 2-group comparisons. *P ≤ 0.05.

We further explored the mechanism by which CXCL1 affects the level of oxidative stress in adipocytes. Firstly, the m^6^A dot blotting assay showed an overall hypomethylation of m^6^A in transcripts after CXCL1 administration in adipocytes (Fig. 7A). To identify potential RNA targets for m^6^A hypomethylation induced by CXCL1, m^6^A-seq analysis showed that most m^6^A binding sites (>80%) were located in the protein-coding sequence (CDS region) and the 3’ noncoding region. The most common motif “GAACG” was remarkably enriched in the m^6^A peaks (Fig. 7B and C). Gene Ontology enrichment analysis further showed that CXCL1 affects adipocyte biological processes such as transcript regulation, protein ubiquitination, mRNA processes, and redox reactions, which mainly occur in the cytoplasm and affect protein binding capacity (Fig. 7D). Kyoto Encyclopedia of Genes and Genomes (KEGG) enrichment analysis showed that CXCL1 leads to influence in phospholipid, mitogen-activated protein kinase (MAPK), and HIF-1 signaling pathways (Fig. 7E). The above results suggest that the alteration of m^6^A methylation level following CXCL1 treatment could affect oxidative stress levels and protein transcription in adipocytes. To further explore the downstream targets involved in m^6^A demethylation, 662 transcripts were found down-regulated (*P* < 0.05, Hypo-down), and 671 transcripts were up-regulated (*P* < 0.05, Hypo-up) when comparing CXCL1-treated adipocytes and the control cells by volcano plot (Fig. 7F). Given the co-occurrence of m^6^A methylation, ROS burst, and mitochondrial fission, the Venn diagrams were used to fish the crucial genes contributing to the biological phenomenon. The results revealed a total of 14 eligible genes including *KAT5*, *BUD23*, *RECQL*, *CSF2*, *APP*, *GTF3C1*, *NCOA3*, *LSM2*, *TFPT*, *RALBP1*, *PALLD*, *NEK7*, *BECN1*, and *KEAP1* (Fig. 7G). The transcript levels of these candidate genes between the 2 groups were shown in a heatmap (Fig. 7H). Of the 14 candidate genes identified, KEAP1 was given priority due to its direct relevance to our findings. Initially, KEAP1/NRF2 signaling is a primary regulator of ROS homeostasis, a process that we have demonstrated to be central to CXCL1-induced lipolysis. Secondly, unlike other candidates with indirect roles, KEAP1 has well-established functions in adipocyte metabolism. Consequently, KEAP1 was selected as the target for demethylation induced by CXCL1.

### CXCL1 regulates *KEAP1* m^6^A demethylation via fat mass and obesity-associated protein

**Fig. 8.**
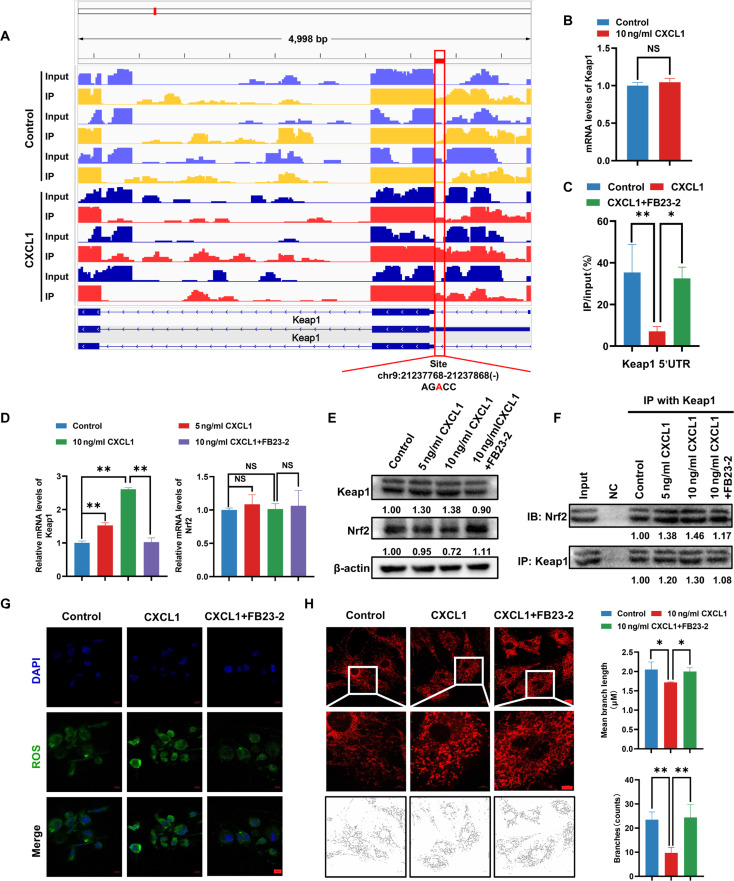
CXCL1 induces KEAP1 m^6^A demethylation to promote oxidative stress and mitochondrial fission in adipocytes. (A) The relative abundance of m^6^A sites along KEAP1 mRNA in CXCL1-treated adipocytes compared to the control group, as detected by m^6^A-seq. The red rectangles indicated that the m^6^A peaks had a markedly decreased abundance. (B) A dual-luciferase reporter assay was applied to detect the effect of CXCL1 on the promoter activity of KEAP1. (C) CXCL1 promoted m^6^A demethylation in KEAP1 mRNA detected by the m^6^A MeRIP analysis. (D and E) KEAP1 and NRF2 mRNA level and protein expression affected by CXCL1 with or without FB23-2 treatment in adipocytes. (F) The protein binding between KEAP1 and NRF2 adipocytes following CXCL1 with or without FB23-2 treatment in adipocytes was detected by Co-IP analysis. Input represents the total protein extracts prepared without the antibody coupling resin. NC indicates the negative control prepared by adding quenching buffer to the antibody coupling resin. (G and H) The level of ROS (G) and mitochondrial fission (H) induced by CXCL1 with or without FB23-2 treatment in adipocytes were detected by immunofluorescence assay. Data are presented as mean ± SD, *n* = 3. Statistical analysis: Data in (B) were analyzed by the Mann–Whitney *U* test. One-way ANOVA with LSD post-hoc test for (C) and (H) and Bonferroni post-hoc test for (D). *P ≤ 0.05, **P ≤ 0.01.

In order to decipher the molecular mechanisms underlying CXCL1-induced *KEAP1* m^6^A demethylation, the demethylation sites of *KEAP1* mRNA are shown in Fig. 8A, which is on the region Chr9: 21,237,768-21237868(AGACC). Meanwhile, dual luciferase reporter gene assay revealed that CXCL1 has little effect on *KEAP1* promoter activity (Fig. 8B), but the m^6^A RNA immunoprecipitation (MeRIP) followed by quantitative polymerase chain reaction (qPCR) assay showed that the level of *KEAP1* 5′ untranslated region (5′UTR) methylation was reduced after CXCL1 treatment in adipocytes, and was partially counteracted by demethylation inhibitor FB23-2 (Fig. 8C). In addition, qPCR and Western blotting results validated that CXCL1 dose-dependently increased both *KEAP1* mRNA and protein expression in adipocytes and was also partially reversed by FB23-2. However, CXCL1 did not influence *NRF2* mRNA expression, but resulted in the down-regulation of NRF2 protein expression (Fig. 8D and E). More importantly, co-immunoprecipitation (Co-IP) results demonstrated that CXCL1 remarkably promoted the binding between KEAP1 and NRF2, which was also recovered by FB23-2 treatment (Fig. 8F). Furthermore, KEAP1-NRF2 binding resulted in proteasome degradation of NRF2 validated by accelerated degradation with protein synthesis inhibitor cycloheximide treatment, but restored expression after administration with proteasome inhibitor MG132 (Fig. S5). Furthermore, it was also found that FB23-2 alleviated CXCL1-induced elevated ROS levels and mitochondrial fission in adipocytes (Fig. 8G and H). Taken together, *KEAP1* m^6^A demethylation is crucial in mediating CXCL1-induced adipocyte lipolysis.

**Fig. 9.**
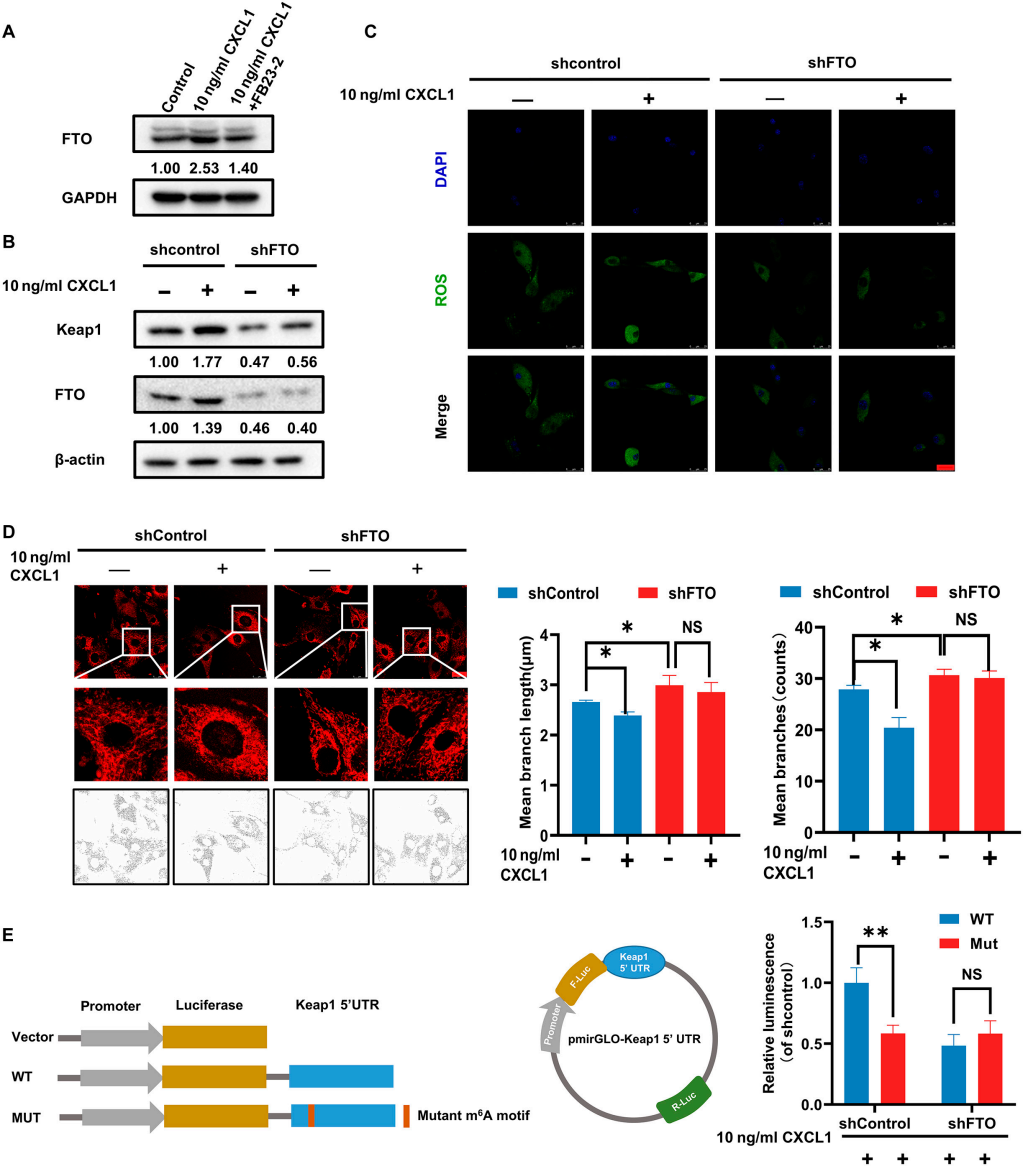
FTO mediates CXCL1-induced KEAP1 m^6^A demethylation and adipocyte dysfunction. (A) FTO expression in adipocytes following 24 h CXCL1 treatment with or without FB23-2. (B) KEAP1 protein expression in control and FTO-knockdown adipocytes with or without CXCL1 treatment. (C) ROS levels detected by DCFH-DA in FTO-knockdown adipocytes following CXCL1 treatment. (D) Mitochondrial fission analysis using MitoTracker in FTO-knockdown adipocytes with CXCL1 treatment. (E) Luciferase activity of wild-type and mutant KEAP1 5′UTR reporters in control and FTO-knockdown adipocytes. Scale bar = 25 μm. Data are presented as mean ± SD, *n* = 3. Student’s *t* test for 2-group comparisons. One-way ANOVA with Bonferroni post-hoc test for (D). *P ≤ 0.05, **P ≤ 0.01.

Fat mass and obesity-associated protein (FTO) is a key enzyme in mediating m^6^A demethylation [34]. Following CXCL1 treatment, FTO expression was notably increased, and FTO knockdown was found to block CXCL1-induced KEAP1 overexpression in adipocytes (Fig. 9A and B). Meanwhile, FTO knockdown also inhibited CXCL1-induced ROS elevation and mitochondrial fission (Fig. 9C and D). To confirm the binding of FTO with the 5′UTR of *KEAP1* mRNA, a luciferase reporting gene vector containing *KEAP1* 5′UTR was constructed. The results showed that either FTO knockdown or the binding site mutation substantially blocked the transcription activity of *KEAP1* 5′UTR. However, in the mutated vectors, FTO knockdown did not influence *KEAP1* transcription activity (Fig. 9E). All these findings suggest that CXCL1 promotes *KEAP1* m^6^A demethylation via FTO.

### CXCL1 knockdown and demethylation inhibitor suppresses CUMS-induced BC growth and metastasis via attenuating adipocyte lipolysis

**Fig. 10.**
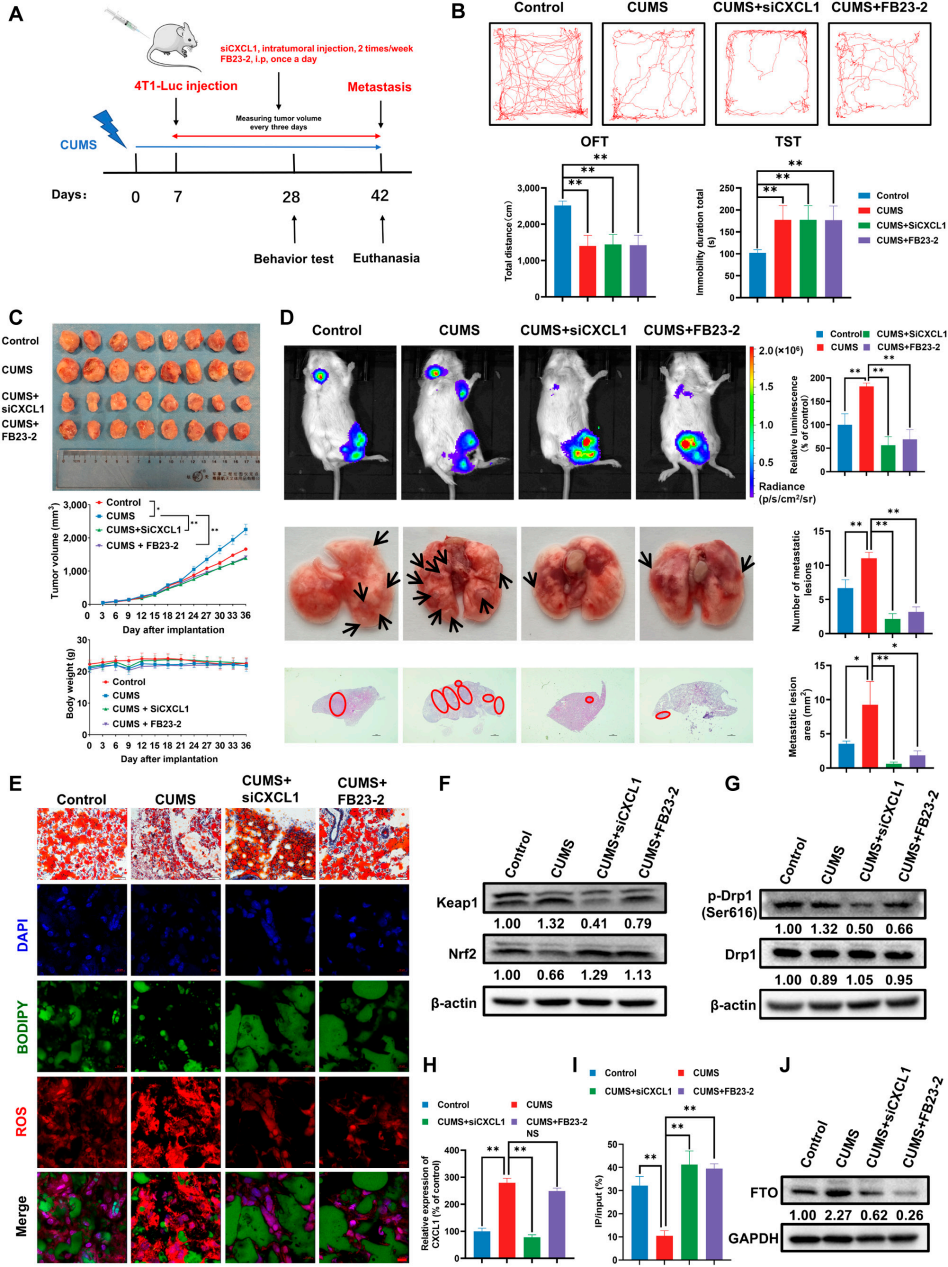
CXCL1 knockdown and demethylation inhibitor FB23-2 suppress CUMS-induced BC growth and metastasis by attenuating adipocyte lipolysis and KEAP1 m6A demethylation. (A) Flowchart of the mouse experiments. (B) Behavioral analysis of OFT and TST (*n* = 6). (C) Representative tumor pictures, BC growth curve and body weight changes in each group (*n* = 8). (D) Assessment of lung metastasis in BC xenograft models. Top panel: In vivo bioluminescence imaging showing metastatic burden. Middle panel: Representative gross lung images with metastatic nodules indicated by arrows. Bottom panel: HE staining of lung sections with metastatic foci marked by red circles. Scale bar = 1 mm. Statistical quantification of metastatic nodule count and area are shown on the right (*n* = 6). (E) Histological analysis of pericancerous adipose tissue. Top panel: Oil Red O staining revealing lipid droplet morphology in adipose tissue adjacent to tumors (*n* = 3). Scale bar = 50 μm. Bottom panels: Dual immunofluorescence staining showing BODIPY (green) for lipid droplets, ROS (red) for oxidative stress, and DAPI nuclear counterstain (blue). Scale bar = 10 μm. (F) The oxidative stress-related protein expression levels in adipose tissue nearby BC were detected by Western blotting. (G) The mitochondrial fission-associated protein levels in adipose tissue nearby BC were detected by Western blotting. (H) The secretion level of CXCL1 in adipose tissue nearby BC of different groups was detected by ELISA (*n* = 3). (I) The m^6^A MeRIP analysis was applied to detect m^6^A demethylation of KEAP1 mRNA in different groups (*n* = 3). (J) The protein expression of FTO in adipose tissue nearby BC was detected by Western blotting. Scale bar = 50 μm. Data are presented as mean ± SD. Statistical analysis: one-way ANOVA with Dunnett *t* post-hoc test for (B); Bonferroni post-hoc test for (D), (H), and (I); and repeated-measures ANOVA for (C). *P ≤ 0.05, **P ≤ 0.01.

We finally confirmed the suppression effects of siCXCL1 and the demethylation inhibitor FB23-2 on CUMS-induced BC growth and metastasis. The flowchart is shown in Fig. 10A. The mice behavior assays demonstrated that the CUMS mouse model was successfully established, and either siCXCL1 or FB23-2 had little effect on the total distance and immobility duration of mice (Fig. 10B). Notably, siCXCL1 and FB23-2 inhibited CUMS-induced BC growth, but had a minor effect on body weight (Fig. 10C). Meanwhile, in vivo imaging assay and ex vivo observation showed that CUMS markedly promoted BC lung metastasis, which was reduced by siCXCL1 and FB23-2 (Fig. 10D). Since the anticancer effects of siCXCL1 and FB23-2 were not related to the improvement of mice depression status, we further detected the changes of para-cancer adipose tissues. The results of Oil Red O staining showed that both siCXCL1 and FB23-2 treatment substantially alleviated the CUMS-promoted lipolysis (Fig. 10E). Moreover, immunofluorescence results of pericancerous adipose tissue showed that both siCXCL1 and FB23-2 inhibited CUMS-elevated ROS levels (Fig. 10E). Western blotting further indicated that siCXCL1 and FB23-2 could inhibit CUMS-induced elevation of KEAP1 and p-DRP1(Ser616) in adipocytes. Correspondingly, the expression of NRF2 was up-regulated by either siCXCL1 or FB23-2 treatment (Fig. 10F and G). In addition, enzyme-linked immunosorbent assay (ELISA) analysis further validated that siCXCL1 inhibited CXCL1 level in the adipose tissue (Fig. 10H), and MeRIP-qPCR results demonstrated that both siCXCL1 and FB23-2 attenuated the elevated KEAP1 demethylation level induced by CUMS (Fig. 10I), accompanied by the reduction of FTO expression (Fig. 10J). All these findings implied that siCXCL1 and FB23-2 could suppress CUMS-mediated BC growth and metastasis via suppressing KEAP1 demethylation-induced adipocyte lipolysis.

## Discussion

Chronic psychological stress has been considered as a crucial risk promoting BC growth and metastasis [35]. Over the past decade, multiple downstream signaling, such as elevated glucocorticoid, altered gut microbiota, enhanced stemness, and suppressed immune microenvironment, have been reported to facilitate CUMS-induced cancer progress [36]. Notably, it has been increasingly recognized that adipocyte lipolysis plays an important role in mediating the growth of solid malignancies, particularly for BC [37]. Meanwhile, a metabolomic study also found that depression is also closely correlated with lipid metabolism. It is therefore interesting to explore whether CUMS could promote BC development via adipocyte lipolysis and the underlying molecular mechanisms. In the present study, it was found that CUMS activated adipocyte lipolysis to promote BC growth and metastasis via TRP-induced TAM/CXCL1 signaling. A molecular mechanism study validated that TAMs/CXCL1 led to *KEAP1* m^6^A demethylation, which subsequently triggered ROS burst, mitochondrial fission, and adipocyte lipolysis. Previously, we also found that CUMS could activate TAM/CXCL1 signaling to promote BC growth and metastasis [36]. However, the upstream factors enhancing TAMs/CXCL1 signaling remain unknown. Consistent with the findings that CUMS increased TRP level in depressive mice model, it was shown that the elevated TRP could activate TAM/CXCL1 signaling and promote adipocyte lipolysis. Our results highlight the important contribution of TRP in mediating adipocyte metabolism and BC development.

TRP is an essential amino acid that is highly implicated in mediating malignant properties and anticancer immunity [38]. Our research uncovered that CUMS impedes the metabolism of TRP, as evidenced by increased TRP concentrations concomitant with diminished levels of its downstream metabolites across the 3 principal metabolic pathways. Several mechanisms might serve as the underlying factors for this paradoxical TRP accumulation. Initially, chronic stress has the potential to compromise the enzymatic activity of TRP-metabolizing enzymes, including indoleamine 2,3-dioxygenase (IDO), tryptophan 2,3-dioxygenase (TDO), and tryptophan hydroxylase (TPH), via oxidative damage, depletion of cofactors, or post-translational modifications, notwithstanding any alterations in enzyme expression [39]. Secondly, the disturbance of the gut microbiota is of paramount importance. It is recognized that CUMS disrupts the gut microbiome, diminishing the population of beneficial bacteria that are actively involved in TRP metabolism. The resultant loss of microbial metabolic function inevitably results in systemic accumulation of TRP [40]. Ultimately, the concurrent inhibition of the 3 principal catabolic pathways implies a comprehensive cessation of metabolism, which may serve as an adaptive mechanism to preserve this vital amino acid during prolonged periods of stress [41,42]. It has been proposed that TRP catabolism is mainly mediated by 3 enzymes including IDO 1/2 and TDO2. IDO1 and TDO2 are the first rate-limiting metalloenzymes that are responsible for the degradation from TRP to kynurenine, which is closely correlated to the failure of anticancer immunotherapies and tumor progression through the activation of AhR [38,43]. With the development of multi-omics techniques, increasing attention has been paid to the role of TRP in the mediation of immunosuppression. A recent metabolomic study revealed that the level of TRP was markedly elevated in BC patients and inhibited IL-10 secretion by CD4^+^ T cells [44]. Consistent with the finding, we also found that TRP level was substantially elevated in mice bearing BC treated with CUMS, and TRP directly increased macrophage M2 polarization, which facilitated the formation of an immunosuppressive tumor microenvironment (TME). Similar to this, it was recently discovered that TAM had high AhR activity, and TRP removal could reduce TAM polarization and promote intratumoral accumulation of TNFα^+^IFNγ^+^CD8^+^T cells [45]. Our research underscores the innovative function and immediate impact of TRP on the mobilization of macrophage polarization, and it is imperative to investigate the role of AhR in this process.

TAMs are a major component in the TME and play a key crucial role in cancer metastasis, immunosuppression, and drug resistance by producing cytokines, chemokines, and growth factors, as well as triggering the release of inhibitory immune checkpoint proteins in T cells [46]. Usually, the abundance of TAM is associated with a poor prognosis in most malignancies [47]. CXCL1 is one of the most abundant chemokines secreted by TAMs and contributes to cancer growth and metastasis [26]. Previous studies demonstrated that CXCL1 was closely correlated with clinical features of multiple malignancies, including poor prognosis, metastasis, reduced survival, and advanced TNM stage, as well as ER, PR, and HER2 status in BC [48]**.** Mechanistically, CXCL1 could recruit various stromal cells into the tumor environment to create a premetastatic niche to facilitate cancer growth, angiogenesis, and metastasis [49]. Intriguingly, macrophage infiltration and the high level of CXCL1 were also enriched in cancer-associated adipocyte tissues, accompanied by an increased number of CLS [50]. Moreover, the CXCL1 receptor CXCR2 was also highly expressed on adipocytes [51]. Mice overexpressing the CXCL1 gene were reported to have markedly less visceral and subcutaneous fat compared with controls, which may be mediated through CXCL1-enhanced FAO [52]. Meanwhile, it was found that CXCL1 is required for obesity-associated adipose stromal cell recruitment, vascularization, and accelerated tumor growth [30]. Consistent with these reports, we also found that the number of CLS was elevated in BC treated with CUMS, accompanied by increased TAMs/CXCL1 signaling. Furthermore, TAMs/CXCL1 could induce adipocyte lipolysis via triggering ROS-mediated mitochondrial fission [19]. Although it has been found that CXCL1 secreted by cancer-associated fibroblasts led to increased ROS burst [53], the underlying molecular mechanisms and their effect on mitochondrial activity are awaiting to be explored. Our study firstly highlights the novel role of CXCL1 in adipocyte lipolysis by elevating ROS level.

ROS is considered as significant intracellular signaling. Intracellular ROS level was precisely controlled by antioxidant systems such as superoxide dismutase, catalases, and peroxiredoxins, as well as glutathione peroxidases [54]. In particular, the KEAP1/NRF2 pathway is well known to balance oxidative stress and participates in various cancerous behaviors such as carcinogenesis, angiogenesis, drug resistance, stemness, and metastasis [55]. KEAP1 is a highly redox-sensitive member of the BTB-Kelch family that assembles with the Cul3 protein to form a Cullin-RING E3 ligase complex for the degradation of NRF2 [56]. Under oxidative stress, NRF2 is released from the NRF2–KEAP1 complex, translocates to the nucleus, and binds to the antioxidant response elements of over 200 genes, leading to the expression of detoxifying enzymes that can neutralize elevated ROS [57]. NRF2 has been well known as the transcript factor of multiple antioxidant genes including HO1, NQO1, GST, and GPX, as well as SOD [58]. Interestingly, accumulating evidence indicates that NRF2 may act as a transcription factor in regulating the formation and activity of adipose tissues, such as adipogenesis, lipid metabolism, and insulin sensitivity [59]. It was shown that *NRF2* knockout in mice reduced adipose tissue mass and prevented high-fat diet-induced weight gain and obesity [60]. Another study also indicated that NRF2 could activate CCAAT/enhancer-binding protein α (C/EBP-α), C/EBP-β, and PPARγ, which are key factors promoting de novo adipogenesis [61]. Besides, mitochondrial fission is a key biological activity in maintaining metabolic homeostasis in both normal physiology and stressful conditions, and ROS plays a vital role in this process [62]. Chang et al. showed that the activated ROS led to mitochondrial fission via Drp1 Ser616 phosphorylation [63]. Consistent with this, our study found that TAMs/CXCL1 inhibited NRF2 expression and resulted in ROS elevation, which subsequently promoted mitochondrial fission and facilitated adipocyte lipolysis. Interestingly, although the *KEAP1* mRNA expression was elevated, its promoter activity was not changed following CXCL1 administration, indicating that the post-transcriptional modification of *KEAP1* might happen.

m^6^A modification is the methylation of the adenosine base at the nitrogen-6 position of mRNA, which is the most pervasive internal modification of mRNA in mammalian cells, with more than 25% abundance in the transcripts [64,65]. The m^6^A modification is mainly mediated by the writer proteins (METTL3, METTL14, and WTAP), eraser proteins (FTO and ALKBH5), and reader proteins (YTH domain family proteins, HNRNPA2B1, and IGF2BP) [34]. The writers, readers, and erasers of m^6^A have been demonstrated to exert crucial effects during cancer occurrence, development, and immunotherapy response through diverse regulation patterns [66]. In the present study, we further investigated whether CXCL1 affects the expression level of *KEAP1* mRNA through FTO regulation. We found that CXCL1 lost its upregulatory effects on *KEAP1* mRNA when FTO was knocked down, and the binding site mutation of *KEAP1* mRNA also blocked the demethylation effects of FTO, suggesting that FTO is the crucial enzyme in promoting *KEAP1* mRNA demethylation, and subsequently accelerates ROS burst, adipocyte lipolysis, and BC growth. Niu et al. [34] also showed that FTO could promote BC development through inhibiting the pro-apoptosis gene BNIP3. Interestingly, another study reported that FTO could facilitate cancer cell escaping from immune surveillance via enhancing glucose metabolism [67]. Moreover, it was also found that FTO targeting suppression could inhibit cancer stem cell maintenance and promote their immune evasion [68]. Compared to the existing reports, we firstly revealed its pro-cancer effects on regulating adipocyte lipolysis via KEAP1/NRF2 signaling. While our data support FTO-mediated KEAP1 demethylation as a key mechanism, we acknowledge that other m^6^A readers and erasers may contribute to the observed effects. Thus, further research is also necessary to identify other m^6^A targets involved in lipolysis by transcriptome sequencing in FTO knockout adipocytes.

The present study is subject to several limitations that merit attention. Initially, while the CUMS model adeptly replicates chronic stress in rodents, it may not entirely capture the intricate nature of human psychological stress, which encompasses cognitive and social elements not represented in animal models. Secondly, our experimental procedures involved immunocompetent BALB/c mice bearing syngeneic 4T1 tumors; confirmation in humanized mouse models and patient-derived xenografts would enhance the clinical applicability. Thirdly, although our research identified FTO as a pivotal m6A demethylase, the possible engagement of additional epitranscriptomic regulators has not been investigated. Subsequent research endeavors should aim to tackle these limitations by employing multi-omics methodologies and conducting clinical validations. In anticipation of future research, it is imperative that clinical trials be conducted to ascertain whether there is a correlation between TRP levels and the stress status as well as treatment outcomes in patients with BC. The therapeutic implications of targeting the TRP/CXCL1/FTO pathway merit further exploration, especially in light of FTO inhibitors currently under development. Moreover, the integration of psychological interventions with metabolic monitoring could facilitate the implementation of precision psycho-oncology approaches.

**Fig. 11.**
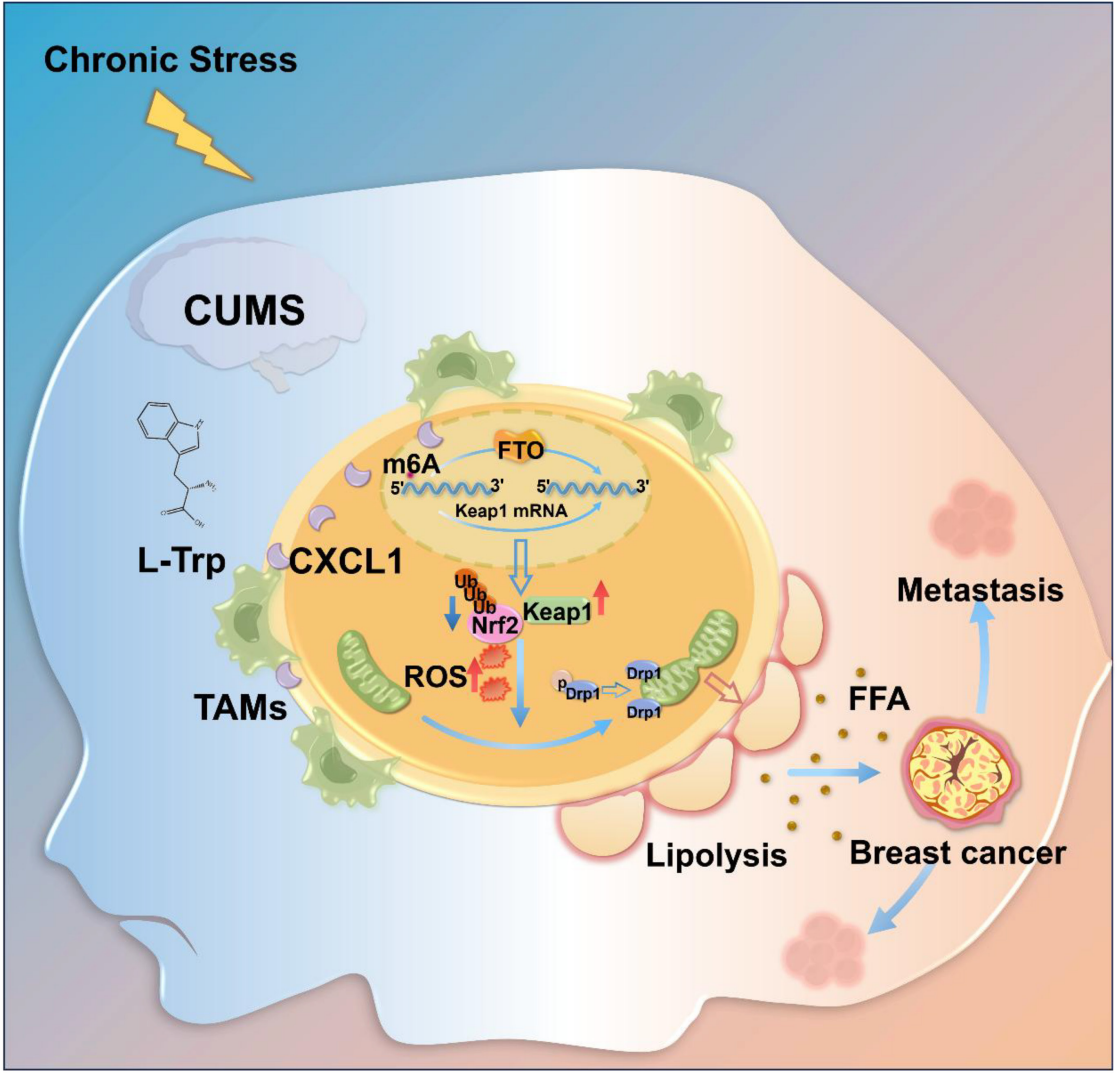
Chronic unpredicted mild stress markedly increases serum TRP levels, thereby activating TAMs/CXCL1 signaling. This signaling leads to KEAP1 m6A demethylation, which, in turn, inhibits NRF2, resulting in adipocyte mitochondrial fission and lipolysis through ROS burst. Subsequently, the FFAs released by lipolysis promote breast cancer growth and metastasis.

Taken together, our study demonstrated that CUMS promoted BC growth and metastasis via enhancing TRP-induced adipocyte lipolysis by activating TAMs/CXCL1/KEAP1 signaling. Our findings not only provide the novel role of TRP in mediating BC adipocyte lipolysis via recruiting TAMs but also uncover the novel mechanisms of *KEAP1* m^6^A demethylation in promoting adipocyte lipolysis via CXCL1-mediated FTO enhancement (Fig. 11).

## Materials and Methods

### Chemicals and reagents

The standards L-tryptophan, xanthurenic acid, kynurenic acid, 3-hydroxyanthranilic acid, 2-picolinic acid, nicotinamide adenine dinucleotide, quinolinic acid, 5-hydroxyindoleacetic acid, indole, indole-3-propionic acid, indoleacetic acid, and indole-3-carboxaldehyde were obtained from Yuanye Biotechnology Company Limited (Shanghai, China). L-kynurenine and melatonin were obtained from Meilun Bio-Technology Co., Ltd. (Dalian, China), and 5-HT was provided by Sigma (San Francisco, CA, USA). Reagent RTA402 was purchased from Selleck (Shanghai, China). CH-223191, Atglistatin (ATGLi), Cycloheximide (CHX), MG-132, and FB23-2 were obtained from MCE (New Jersey, USA).

### Animal experiments

Female BALB/c mice (5 weeks old) were supplied by the Experimental Animals Center of Guangdong Province (Guangzhou, China) and kept in a standard pathogen-free ventilated room. The Institutional Animal Care and Use Committee of Guangdong Provincial Hospital of Chinese Medicine approved all mice experimental procedures, and guidelines establishing humane endpoints were rigorously followed (Ethical approval number: 2023075). To establish the CUMS model, 8 kinds of stress were applied including 4 h restraint stress, 24 h food abstinence, 24 h water cutoff, 24 h cage tilt (45°), 5 min forced swimming (4 to 8 °C) water, 12 h diurnal reversal, 15 min tail clamping, and moist bedding for 24 h. Two different stressors were carried out each day at random. After 1 week administration, 2 × 10^6^ 4T1-Luc cells were injected into the fourth mammary fat pads of mice to establish the orthotopic BC model. Mice were subsequently grouped including control and CUMS (*n* = 8). The open field test (OFT) and TST were conducted on the 28th day. Tumor volume measurement was performed every 3 days, followed by calculation using the formula ([width]^2^ × [length]/2). The IVIS-Spectrum system (PerkinElmer, Boston, USA) was used to monitor tumor growth and lung metastasis by intraperitoneally injecting 150 mg/kg of D-luciferin (PerkinElmer, Boston, USA). For the in vivo assay of anticancer effects of siCXCL1 or FB23-2, mice were randomly grouped including control, CUMS, CUMS+siCXCL1, and CUMS+FB23-2. siCXCL1 was administered by intratumoral injection at 1 nM every 3 days. FB23-2 was given by intraperitoneal injection at 2 mg/kg/day. Tumor growth was monitored as stated above. To minimize the selection bias and confounders, a computer-based random number generator for comprehensive randomization management was utilized in the study for the sequence of treatments and measurements, as well as the animal/cage location, thereby maintaining the integrity of the study outcomes.

### Behavioral analysis

For the OFT assay, after being placed individually in the field center (40 × 40 × 40 cm), mouse trajectories were recorded continuously using a video camera for 6 min. For TST detection, mice were fixed with tape about 1 cm from their tail tips and suspended 45 cm above the ground, and their immobility time and latency were recorded for 6 min. Smart 3.0 (PanLab, Cornella, Spain) was used to analyze all captured videos.

### Metabolomic study

Mouse serum (80 μl) was added with 240 μl of methanol for protein removal. After incubation on ice for 30 min, the mixture was centrifuged (4 °C, 12,000 r/min), and the supernatant was separated and blown dry under nitrogen. Finally, 80 μl of 50% acetonitrile solution was added and filtered with 0.22-μm filters. An Ultimate 3000 HPLC and a Q Exactive Plus mass spectrometer (Thermo Fisher Scientific) were employed for TRP metabolite analysis. The chromatographic separation was carried out using an Acquity UPLC BEH C18 column (1.7 μm, 100 × 2.1 mm; Waters). The mobile phase consisted of solvent A (0.1% formic acid in water) and solvent B (acetonitrile). The LC time program was set as follows: 5% solvent B (0 to 1 min), 5%–100% solvent B (1 to 29 min), 100%–5% solvent B (29 to 30 min), and 5% solvent B (30 to 34 min), with 200 μl/min flow rate, 5 μl injection volume, and 37 °C column temperature. Mass spectrometer settings were determined as spray voltage (2.5 kV, negative mode; 3.0 kV, positive mode), 40 sheath gas, 10 auxiliary gas, and 325 °C capillary temperature setting. Full scan resolution was 70,000, and acquisition range was 100 to 1,000 (*m*/*z*). The automatic gain control target was regulated at 1 × 10^6^. Xcalibur 4.0 software (Thermo Fisher Scientific) was employed for data acquisition and analysis.

The raw LC-MS/MS data files were analyzed utilizing Compound Discover 3.1 (Thermo Fisher Scientific) to obtain the matched and aligned peak data. After normalization in Excel, the peak area data were imported into SIMCA-P 14.1 (Umetrics, Sweden) for PCA and partial least squares discriminant analysis (PLS-DA). KEGG pathway enrichment was analyzed utilizing metaboanalyst 5.0 software (https://www.metaboanalyst.ca/). All identified metabolites were in accordance with the Standard Initiative for Metabolomics.

### Cell culture

The 4T1 cell line, Raw264.7, and mouse preadipocytes 3T3-L1 were supplied by Kaiji Biotechnology Co., Ltd. (Nanjing, China). 4T1-Luc cells were generated with a lentiviral vector carrying the luciferase gene. The 4T1, 4T1-Luc, and 3T3-L1 cells were maintained in Dulbecco’s Modified Eagle Medium (DMEM) (Gibco, NY, USA) with 10% fetal bovine serum and 1% penicillin–streptomycin (Gibco, NY, USA), and Raw264.7 cells were cultured in RPMI 1640 complete culture medium. For adipocyte differentiation, 3T3-L1 preadipocytes underwent 48 h induction with DMEM complete medium containing 1 μM dexamethasone (HY-14648, MCE, New Jersey, USA), 0.5 mM 3-isobutyl-1-methylxanthine (IBMX) (HY-12318, MCE, New Jersey, USA), and 10 μg/ml insulin (PB180432, Procell, Wuhan, China). Next, the medium was replaced with DMEM complete medium containing 10 μg/ml insulin for another 48-h culture. Finally, DMEM complete medium was used. After 8 to 10 days of induction, more than 90% 3T3-L1 cells presented adipocyte phenotype filled with lipid droplets.

For primary adipocyte culture, the pericancerous adipose tissue was cut into small pieces and digested with 0.15% type II collagenase at 37 °C for 30 min. After centrifugation at 2,000 rpm, the upper suspension cells were passed through a 200-μm filter and cultured in DMEM.

### Conditioned media preparation

All conditioned media (CM) from adipocytes were collected when 3T3-L1 cells were fully differentiated. After treatment with CXCL1, IBMX, or ATGLi for 6 h, the medium of adipocytes was replaced with DMEM. CM from adipocytes was harvested after 6 h, followed by centrifugation at 1,000 rpm for 5 min. The supernatants were finally filtered and applied to 4T1 cells.

To obtain CM for macrophages, macrophages treated with or without 200 μM L-Trp were washed with fresh RPMI 1640 medium and incubated for another 24 h. The cell-free supernatant was harvested and subjected to centrifugation at 1,000 rpm for 15 min, followed by filtering through 0.22-μm filters and put into use.

### Oil Red O staining and BODIPY staining

For lipid staining, fresh pericancerous fat samples were embedded in optimal cutting temperature compound (Sakura, Torrance, USA) and sectioned at 10 μm. The sections were stained with Oil Red O solution (3 mg/ml, Solarbio, Beijing, China) for 30 min, followed by nuclei restaining with hematoxylin (Yongjin, Guangzhou, China) for 1 min. Finally, the sections were sealed with glycerol gelatin (Beyotime Biotechnology, Shanghai, China). For 3T3-L1 adipocytes staining, 4% paraformaldehyde was used to fix the cells for 20 min, and Oil Red O was used for staining for 30 min at room temperature. The images were captured with a BX61 microscope (Olympus, Center Valley, PA, USA). With regard to BODIPY staining, the frozen sections of pericancerous adipose tissue were firstly fixed in 4% paraformaldehyde for 20 min. After washing thrice with phosphate-buffered saline (PBS), 2 μM BODIPY (D3922, Invitrogen, Carlsbad, CA, USA) was added and incubated for 30 min at 37 °C, followed by 10 min staining with 4′,6-diamidino-2-phenylindole (DAPI) (C1006, Beyotime Biotechnology, Shanghai, China). The sections were sealed with an antifade mounting medium and imaged using the LSM710 confocal microscope (Zeiss, Jena, Germany).

### ROS detection

Cellular ROS was detected using the fluorescence probe 2,7-dichlorofluorescin-diacetate (DCFH-DA) (S0033S, Beyotime Biotechnology, Shanghai, China). After 20-min incubation with 10 μM DCFH-DA away from light at 37 °C, cell nuclei underwent staining with DAPI and were observed under a laser-scanning confocal microscope or quantified by flow cytometry. For tissue ROS determination, frozen pericancerous adipose tissue sections were incubated with dihydroethidium (D11347, Invitrogen, Carlsbad, CA, USA) at 37 °C for 30 min. The tissues were photographed under an LSM710 confocal microscope.

### Immunohistochemistry and HE staining

The immunohistochemical assay was performed by the Polymer Detection System (PV-9000, Zhongshan Jinqiao Biotechnology) following the manufacturer’s instructions. The primary antibodies against CD68 (DF7518, Affinity Biosciences, Cincinnati, OH, USA) and peroxidase-conjugated goat anti-rat IgG (ZB-2307, Zhongshan Jinqiao Biotech) were used. HE staining analysis was performed as previously reported [69]. Briefly, tissue sections were dewaxed, followed by staining with 10% hematoxylin and 1% eosin (Yongjin, Guangzhou, China). The sections were finally dehydrated and transparent before being sealed with neutral gum. A BX61 microscope (Olympus, Center Valley, PA, USA) was utilized for digital image capture.

### Lipolysis measurement

Lipolysis was assessed by measuring the levels of glycerol and NEFA released into the medium. After 6 h of treatment, NEFA and glycerol release in the medium were respectively measured with an FFA assay kit and a glycerol content test kit according to the manufacturer’s instructions (A042-2-1/F005-1-1, Nanjing Jiancheng, China).

### Flow cytometry assay

Macrophage polarization in pericancerous adipose tissue and TRP-induced Raw264.7 polarization were detected using the NovoCyte Quanteon Flow cytometer (Agilent Technologies, California, USA). Antibodies applied for cell analysis include APC-F4/80 antibody (17-4801-80, eBioscience, San Diego, CA, USA) and PE-CD206 antibody (555954, BD Biosciences, San Diego, CA, USA).

### ELISA assay

CXCL1 concentrations in TRP-treated Raw264.7 cell culture supernatants and in pericancerous adipose tissue were measured using the Mouse CXCL1 ELISA Kit (SEA041Mu, USCN Business) following the manufacturer’s instruction.

### Western blot and immunoprecipitation

Western blotting assays were performed as previously reported [69]. The primary antibodies included CXCL1 (12335-1-AP), DRP1 (12957-1-AP), OPA1 (27733-1-AP), NFE2L2 (16396-1-AP), α-tubulin (11224-1-AP), Lamin B1(12987-1-AP), Keap1 (10503-1-AP), FTO (27226-1-AP) (Proteintech, Rosemont, IL, USA), Phospho-HSL (Ser660) (AF8026), HSL (AF6403), ATGL (DF7756), Phospho-DRP1(Ser637) (DF2980), Phospho-DRP1(Ser616) (AF8470) (Affinity Biosciences, Cincinnati, OH, USA), and β-actin (4970S, Cell Signaling Technology, Boston, MA, USA). A Pierce Co-Immunoprecipitation Kit (no. 26149, Thermo Fisher Scientific, Hudson, NH, USA) was applied for immunoprecipitation assay as directed by the manufacturer’s protocol. Primary antibodies for KEAP1 and NFE2L2 were used in this assay. The relative protein expression level between groups was determined with band optical densities by Gel-Pro analyzer 4 (Media Cybernetics, USA).

### Immunofluorescence assay

Fresh tissue sections or fixed cells were incubated with the primary antibodies including NFE2L2 (16396-1-AP, Proteintech, Rosemont, IL, USA) and phospho-DRP1 (Ser616) (AF8470, Affinity Biosciences, Cincinnati, OH, USA) at 4 °C overnight, followed by blocking in 5% bovine serum albumin for 30 min. The fluorescence-conjugated secondary antibody was then incubated away from light for 1 h at room temperature. DAPI was employed for nuclei visualization under an LSM710 confocal microscope.

### Mitochondrion imaging analysis

Mitochondrion shape was observed utilizing transmission electron microscopy. Briefly, adipocytes were fixed with electron microscopy fixative at 4 °C overnight, followed by PBS washing and osmium fixation for 2 h. After dehydration with an ethanol gradient, the sections were embedded and sectioned with an ultrathin microtome (LEICA EM UC7, Wetzler, Germany). Finally, thin sections were stained with uranyl acetate and lead citrate and analyzed using an electron microscope (JEOL JEM-1200EX, Tokyo, Japan). For mitochondrial fission analysis, the mitochondrial morphology was monitored using fluorescent dyes. For live-cell imaging, MitoTracker Red FM (M22425, Molecular Probes, Eugene, OR, USA) was used according to the manufacturer’s protocol. For fixed cells, MitoTracker Red CMXRos (M7512, Molecular Probes, Eugene, OR, USA) was used, which is retained in mitochondria even after aldehyde fixation and detergent permeabilization. A laser-scanning confocal microscope was utilized for cell visualization. The length and number of all mitochondrial branches in each cell were measured using the Mitochondrial Network Analysis (MiNA, https://github.com/StuartLab/MiNA) macro in Fiji ImageJ [70].

### RNA m^6^A dot blot assay

Immobilon-Ny+ nylon membrane (INYC00010, Merck Millipore) was spotted with poly (A) RNAs (100 or 300 ng). After being ultraviolet cross-linked (254 nm) and blocked with 5% nonfat dry milk for 2 h, the membrane was incubated overnight with m^6^A antibody (1:250 dilution, ab286164, Abcam) at 4 °C. Next, the horseradish peroxidase-conjugated goat anti-rabbit IgG (A00208, Beyotime Biotechnology, Shanghai, China) was dropped in the blots and incubated for 1 h at room temperature. Subsequently, 0.2% methylene blue in 0.3 M sodium acetate (pH 5.2) was used to further stain the nylon membrane for 2 h, followed by rinsing in ribonuclease-free water for 1 h. The membrane was finally exposed in the visualizer (5200, Tanon, China).

### m^6^A-seq assays and data analysis

Total RNA was extracted from CXCL1-treated adipocytes and control adipocytes using Trizol (Invitrogen, Carlsbad, CA, USA). Approximately more than 1 μg of total RNA was fragmented utilizing Magnesium RNA Fragmentation Module (NEB, catalog no. e6150, USA) at 94 °C for 5 min. Next, the cleaved RNA fragments (including rRNA fragments) were incubated with m^6^A antibody-dynabeads compounds (m^6^A IP). The IP RNA was used to build the final cDNA library with a fragment size of 300 ± 50 bp after 2 rounds of PCR. Finally, the 2 × 150-bp paired-end sequencing (PE150) was performed on an Illumina Novaseq 6000 (LC-Bio Technology CO., Ltd., Hangzhou, China) following the protocol.

For data analysis, the clean reads were acquired utilizing the fastp software (https://github.com/OpenGene/fastp). HISAT2 (http://daehwankimlab.github.io/hisat2) was used to map reads to the reference genome Mus musculus (Version: v101). Mapped reads of IP and input libraries were provided for the R package exomePeak2 (https://bioconductor.org/packages/release/bioc/html/exomePeak2.html), which identifies m^6^A peaks that can be visualized using the IGV software (http://www.igv.org). The R package ANNOVAR (http://www.openbioinformatics.org/annovar/) was employed for peak annotation. Motif analysis was conducted utilizing MEME (http://meme-suite.org) and HOMER (http://homer.ucsd.edu/homer/motif). The gene assembly and quantification software was StringTie (https://ccb.jhu.edu/software/stringtie), and the quantification method was FPKM [total exon fragments /mapped reads (millions) × exon length (kB)]. Differentially expressed transcripts and genes were screened with log2 (fold change) ≥ 1 or log2 (fold change) ≤ −1 and *P* value < 0.05 by the R package edgeR (https://bioconductor.org/packages/edgeR).

### MeRIP and MeRIP-qPCR

MeRIP was performed utilizing a commercially available ribo*MeRIP* m^6^A Transcriptome Profiling Kit (C11051-1, RIBOBIO, Guangzhou, China) according to the kit instructions. In summary, total RNA was extracted by Trizol reagent (AG21101, Accurate Biology, Changsha, China). RNA was randomly fragmented using RNA fragmentation reagents. The anti-m^6^A antibody was used for m^6^A immunoprecipitation. Anti-m^6^A antibody was prebound to magnetic beads A/G in 1× IP buffer for 30 min. The fragmented mRNA was incubated with m^6^A-antibody-bound magnetic beads A/G at 4 °C for 2 h and washed with 1× IP buffer. RNA bound to m^6^A antibody was extracted from the magnetic beads using elution buffer. BeyoMag RNA Clean Magnetic Beads (R0081, Beyotime Biotechnology, Shanghai, China) were used to purify the eluted RNA. The RNA and Clean Magnetic Beads were incubated at room temperature for 5 min, and the mixture was rinsed with 80% ethanol and resuspended in nucleic acid-free water. The beads were pulled to the side of the tube by a magnetic field, and the supernatant was then carefully collected. Reverse transcription polymerase chain reaction was carried out following m^6^A-IP to assess the target gene for m^6^A methylation. The primers for 5′UTR of murine *KEAP1* were designed as follows: 5′-ACAGTTCCTCTGCTAGCCGTTAG-3′ (forward) and 5′-TTTGGTTGCATAGTGAGTTGGAAGCC-3′ (reverse).

### qPCR assay

The qPCR assay was performed based on our previous report [71]. The primer sequences of murine *β-ACTIN*, *KEAP1*, and *NRF2* were listed as follows:

5′-GGAGGGGGTTGAGGTGTT-3′ (*β-ACTIN* forward),

5′-GTGTGCACTTTTATTGGTCTCAA-3′ (*β-ACTIN* reverse);

5′-TGCCCCTGTGGTCAAAGTG-3′ (*KEAP1* forward),

5′-GGTTCGGTTACCGTCCTGC-3′ (*KEAP1* reverse);

5′-TCTTGGAGTAAGTCGAGAAGTGT-3′ (*NRF2* forward),

5′-GTTGAAACTGAGCGAAAAAGGC-3′ (*NRF2* reverse).

### Plasmid transfection

The commercialized recombinant shRNA plasmids targeting *FTO* and the nontarget control plasmids (Vigene Biosciences, Jinan, China) were transfected utilizing Vigene fection (FH880806, Vigene Biosciences) according to the instruction.

### Luciferase reporting assays

The *KEAP1* promoter fragment was amplified and cloned into the pEZX-LvPG04 vector (GeneCopoeia, Rockville, MD, USA) carrying the Gaussia luciferase (GLuc) and secreted alkaline phosphatase (SEAP) reporter genes. The *KEAP1* promoter-luciferase reporter vector was introduced into adipocytes. The luciferase activity in the supernatant was detected utilizing a secreted bioluminescence kit (LF001, GeneCopoeia, Rockville, MD, USA). Luciferase activity was normalized by SEAP activity. Meanwhile, the wild-type and mutant *KEAP1*-5′UTR (m^6^A was replaced by T) fragments (Vigene Biosciences) were inserted into the pmirGLO firefly luciferase vector, respectively. Following 48 h post-transfection, the supernatant was harvested for luciferase activity determination.

### Statistical analysis

SPSS 26.0 was employed for data analysis. Student’s *t* test or one-way analysis of variance (ANOVA) was applied, in which the least significant difference (LSD) method was employed for comparisons between 3 groups, the Bonferroni method was used for comparisons between more than 3 groups, and the Dunnett *t* test was used for comparisons between the experimental and control groups. Data without normal distribution were analyzed utilizing the nonparametric test. Repeated-measures ANOVA was utilized for repeated measurement data. Regarding blinding, only the personnel directly involved in conducting the experiments were aware of the group assignments and other experimental manipulations. This information was strictly concealed from individuals responsible for outcome assessments and statistical analysis. *P* < 0.05 was indicated as a statistically significant difference. GraphPad Prism 8.0 software was utilized for plotting.

## Data Availability

All data needed to evaluate the conclusions in the paper are present in the paper and/or the Supplementary Materials.
