# Supplementary material for: Chronic Psychological Stress Activates TRP/TAM/CXCL1 Signaling to Promote Breast Cancer Adipocyte Lipolysis via KEAP1 m6A Demethylation
Source: Research (Wash D C). 2025 Nov 17;8:0980. doi: 10.34133/research.0980 (PMC12620626; doi:10.34133/research.0980)
Supplement: Supplementary 1 — Figs. S1 to S5 [file research.0980.f1.zip › Supplementary Figures-V3.docx]

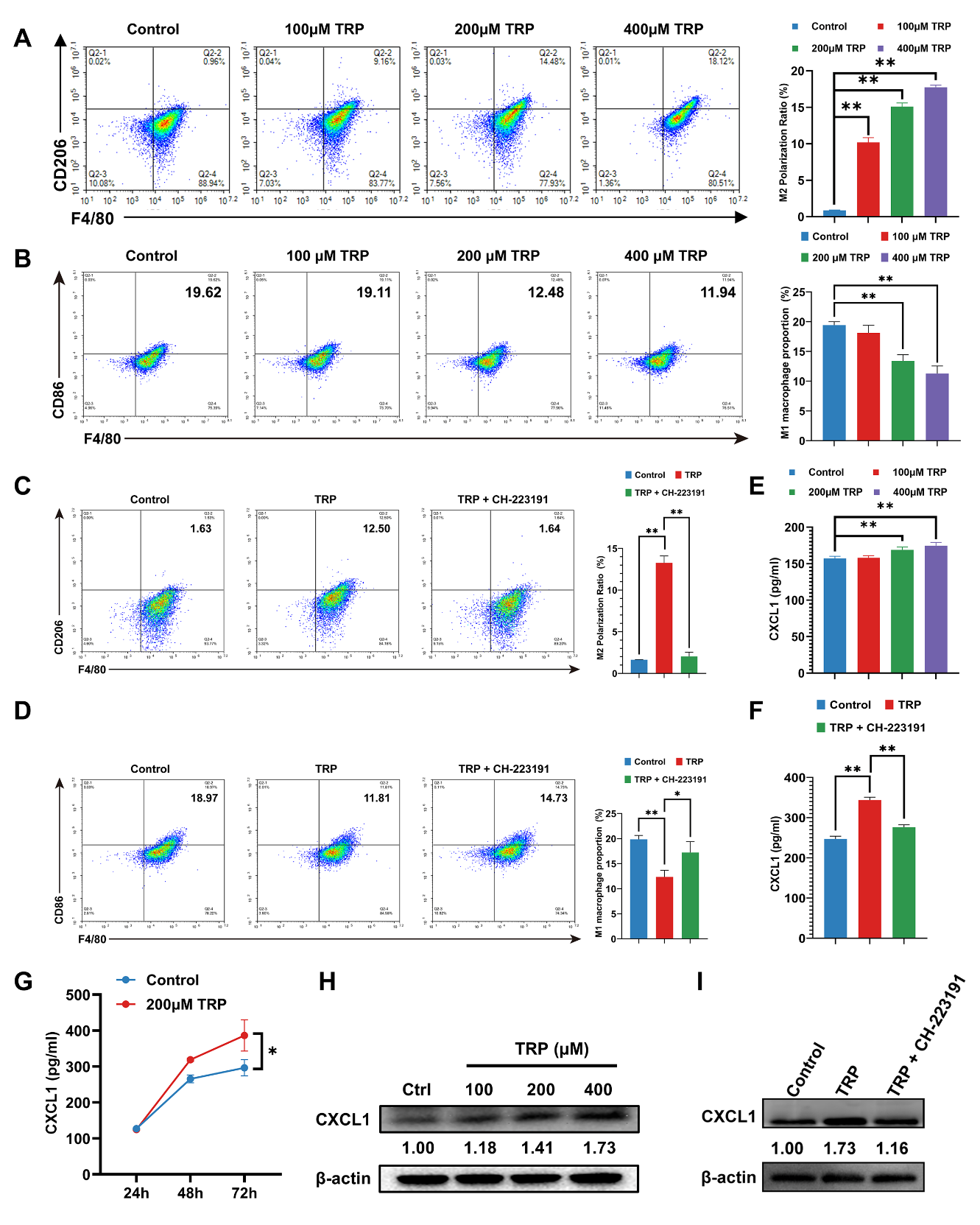


**Figure S1.** **TRP promotes M2 polarization of macrophages and induced CXCL1 expression.** (A-D) Flow cytometry assay was conducted to investigate the effect of TRP treatment (100-400 μM) with or without CH-223191 (10 μM) on the M2 and M1 polarization of Raw264.7 macrophages. (E) Dose-dependent CXCL1 secretion in Raw264.7 macrophages following TRP treatment (0-400 μM) detected by ELISA. (F) CXCL1 secretion following TRP treatment with or without AhR antagonist CH-223191. (G) Time-dependent CXCL1 secretion in Raw264.7 macrophages following treatment with 200 μM TRP. (H-I) The expression level of CXCL1 in Raw264.7 macrophages was detected by western blotting after 24 h of TRP with or without CH-223191 treatment. Data are represented as mean ± SD, n=3. Statistical analysis: one-way ANOVA with LSD post-hoc test for (C, D, F), Dunnett-t post-hoc test for (A, B, E), and repeated measures ANOVA for (G). *P ≤0.05, **P ≤0.01.


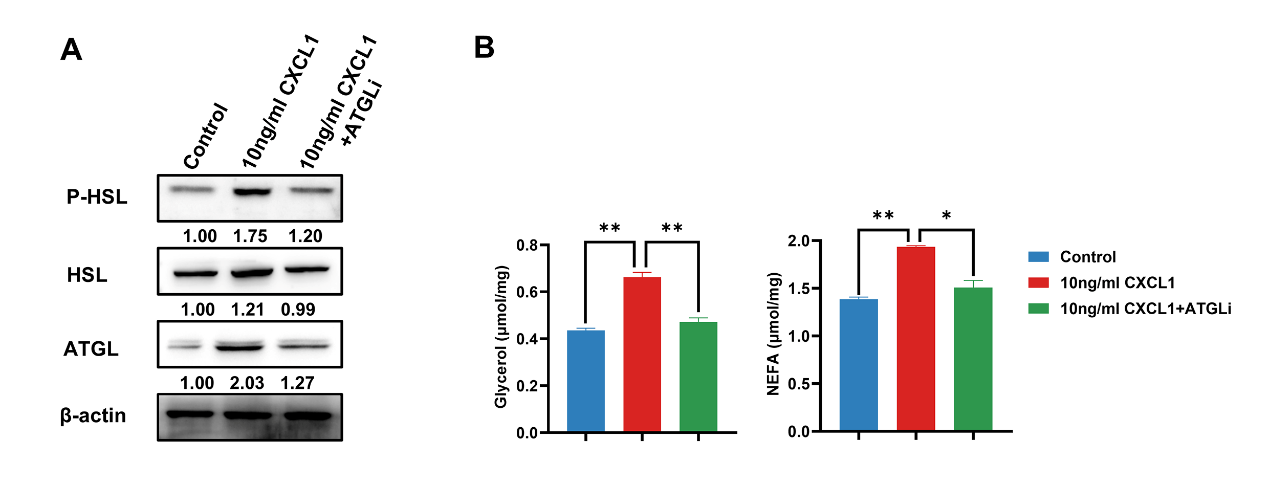


**Figure S2.** **CXCL1 promotes lipolysis in adipocytes.** (A) The lipolysis-related protein expression levels were detected by western blotting after CXCL1 treatment with or without 40 μM ATGLi treatment at 24 h. (B)The glycerol and NEFA levels in culture medium after CXCL1 treatment with or without 40 μM ATGLi at 6 h. Data are represented as mean ± SD, n=3. Statistical analysis: one-way ANOVA with LSD post-hoc test for comparisons between three groups. **P* ≤*0.05, **P* ≤*0.01.*


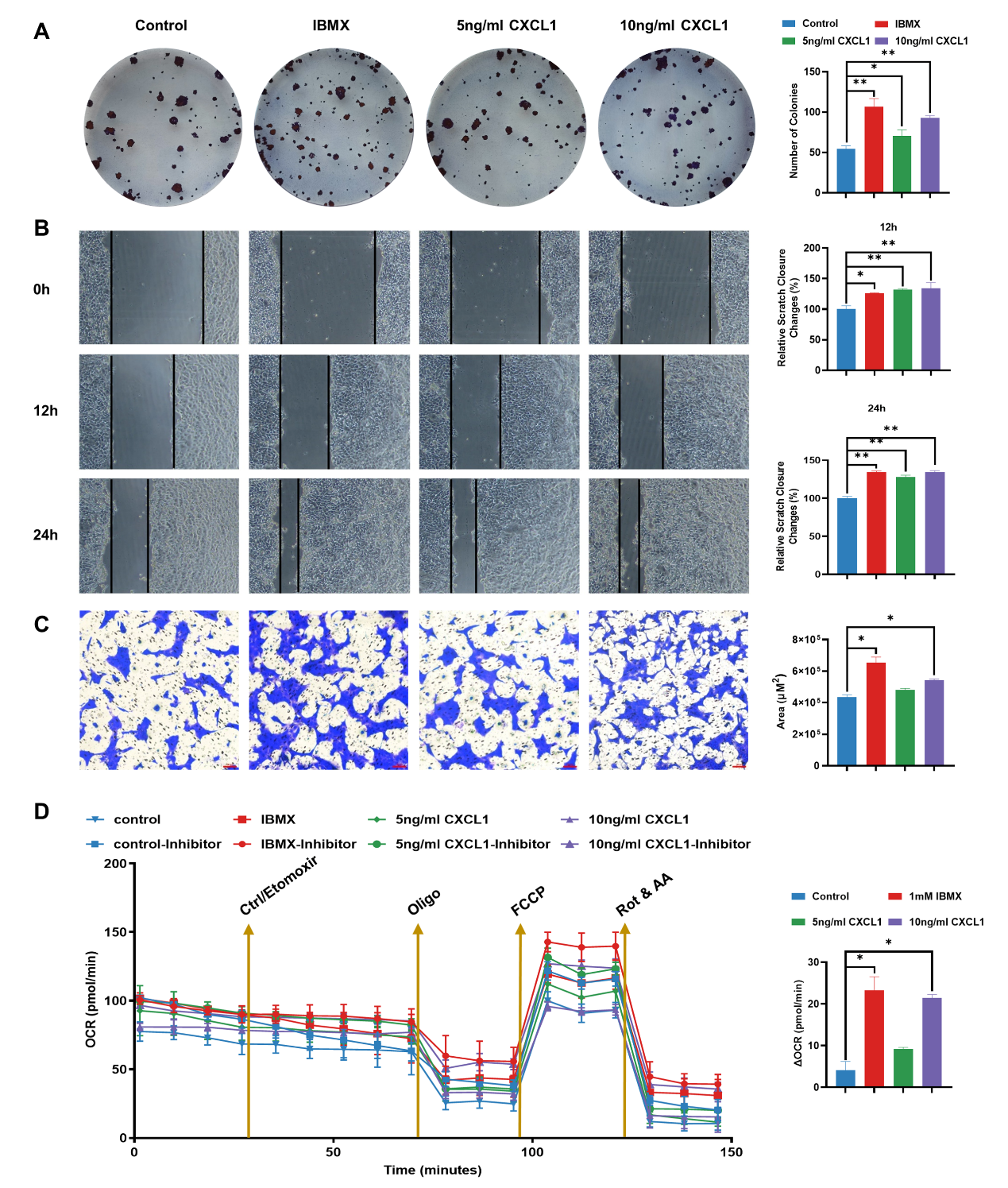


**Figure S3.** CXCL1-mediated lipolysis promotes breast cancer cell growth and metastasis. (A-C) Colony formation (A), wound healing (B), and transwell invasion (C) of 4T1 cells cultured in the medium of adipocytes treated with CXCL1 or IBMX. Scale bars represent 50 μm for transwell assay images. (D) Following culture in the medium of CXCL1/IBMX-treated adipocytes for 24 h, the oxygen consumption rate (OCR) of 4T1 cells was measured using an extracellular flux analyzer. Data are represented as mean ± SD, n=3. Statistical analysis: one-way ANOVA with Dunnett-t post-hoc test was used. **P* ≤0.05, ***P* ≤0.01.


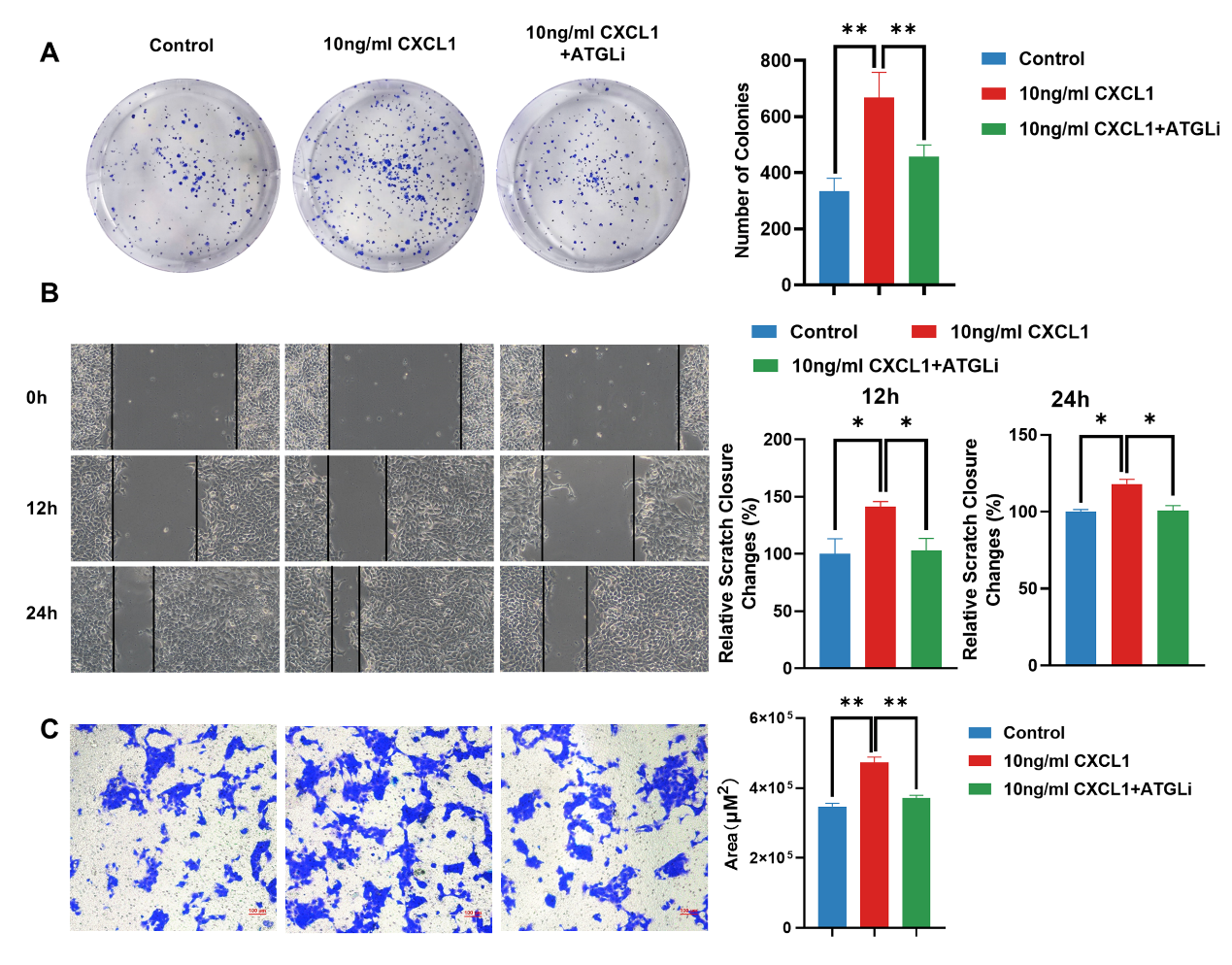


**Figure S4.** CXCL1 promotes breast cancer cell growth and metastasis by inducing lipolysis in adipocytes. (A-C) Colony formation (A), wound healing (B), and transwell invasion (C) of 4T1 cells cultured in the medium of adipocytes treated with CXCL1 and 40 μM ATGLi. Scale bars represent 50 μm for transwell assay images. Data are represented as mean ± SD, n=3. Statistical analysis: one-way ANOVA with LSD post-hoc test for comparisons between three groups. **P* ≤0.05, ***P* ≤0.01.


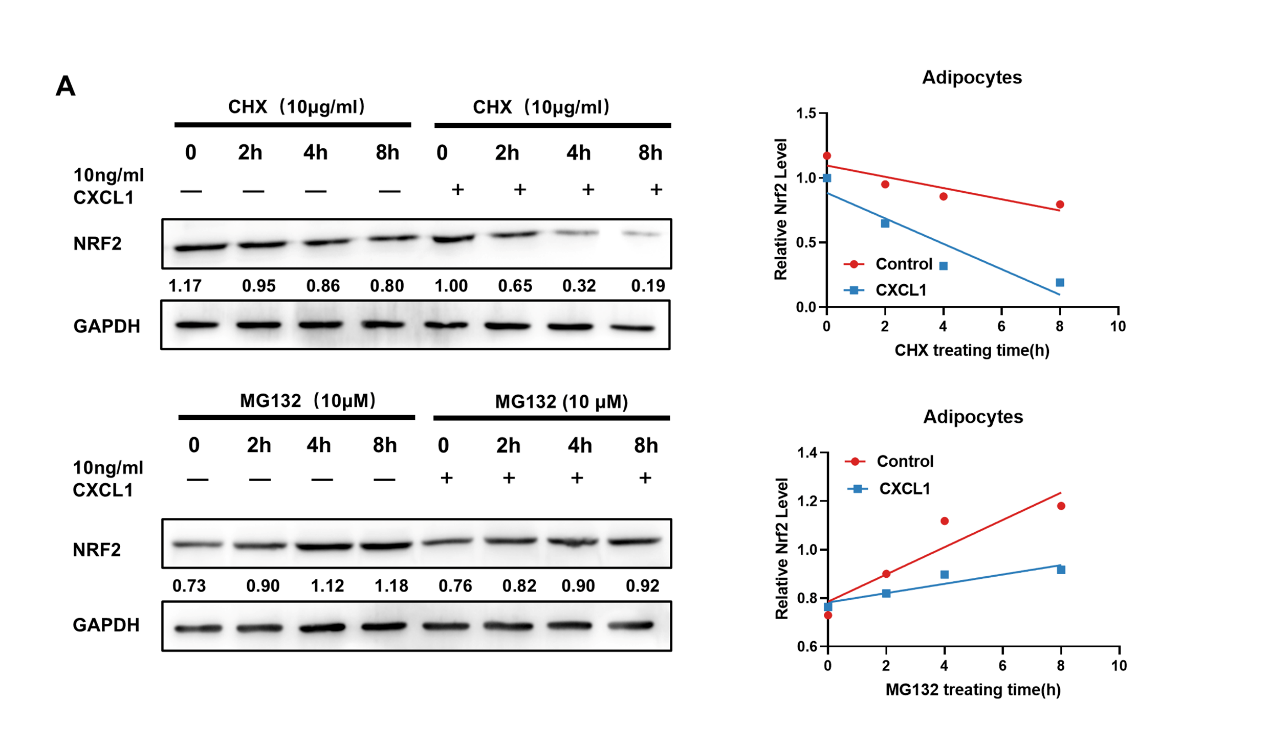


**Figure S5**. **CXCL1 promotes NRF2 proteasomal degradation.** (A) The NRF2 expression levels were detected by western blotting after CHX/MG132 treatment with or without CXCL1 at the indicated time points.
