# Supplementary material for: Chronic Psychological Stress Activates TRP/TAM/CXCL1 Signaling to Promote Breast Cancer Adipocyte Lipolysis via KEAP1 m6A Demethylation
Source: Research (Wash D C). 2025 Nov 17;8:0980. doi: 10.34133/research.0980 (PMC12620626; doi:10.34133/research.0980)
Supplement: Supplementary 1 — Figs. S1 to S5 [file research.0980.f1.zip › Supplementary methods.docx]

**Supplementary methods**

**Colony formation assay**

The effects of CXCL1-mediated lipolysis on colony formation ability of 4T1 cells were investigated using a colony formation assay. 4T1 cells were seeded in 6-well plates at a density of 500 cells per well. After attachment, cells were treated with the culture medium (CM) from CXCL1/IBMX-treated adipocytes. The resultant colonies were fixed with 4% paraformaldehyde and stained with a 0.1% Coomassie blue solution.

**Wound healing and transwell assay**

For the wound healing assay, 4T1 cells treated with CM of CXCL1/IBMX-treated adipocytes grown in 6-well plates as confluent monolayers were mechanically scratched using a 1 ml pipette tip at 0 h to create the wound. Subsequently, the cells were washed twice with PBS and refreshed with complete medium. Wound gaps were microscopically recorded at 12 h/24 h, and the wound gap distances were compared among the groups. For the transwell assay, 100 μl Matrigel (354248, Corning, NY, USA) was smoothly spread on the upper chamber (8 μm pore size, Millipore, Billerica, MA, USA) of the 24-well transwell insert to simulate the basement membrane, and then incubated overnight at 37 °C for gelling. Subsequently, 4T1 cells, which were treated with CM from CXCL1/IBMX-treated adipocytes, were serum-starved for 24 h and then seeded in Matrigel-coated chambers at a density of 1 × 104 cells/chamber in serum-free medium. The lower chamber of the transwell inserts was filled with 600 μl complete medium. The invasive cells were fixed, stained with Coomassie blue after 24 h, and counted using ImageJ.

**Oxygen consumption rate (OCR) measurement**

The XF24 extracellular flux analyzer (Seahorse Bioscience, Billerica, USA) was used to detect real-time changes in the extracellular acidification rate (ECAR) and oxygen consumption rate (OCR). Briefly, cells treated with CM from CXCL1/IBMX-treated adipocytes were seeded at a density of 4 × 104 cells per well and cultured overnight. The cells were then treated with different concentrations of BA or 50 µM 3-BrPA (CAS:1113-59-3, purity: 97%, Sigma-Aldrich) for 3 h prior to measurement.

For OCR analysis (Cell Mito Stress Test), 1 µM oligomycin, 1 µM carbonyl cyanide 4-(trifluoromethoxy) phenylhydrazone (FCCP), 0.5 µM rotenone and 0.5 µM antimycin A (Agilent) were added. All measurements were performed according to the manufacturer’s instructions and normalized to the number of cells counted in each well at the end of the seahorse experiments.
